# Two novel regulators of *N*‐acetyl‐galactosamine utilization pathway and distinct roles in bacterial infections

**DOI:** 10.1002/mbo3.307

**Published:** 2015-11-05

**Authors:** Huimin Zhang, Dmitry A. Ravcheev, Dan Hu, Fengyu Zhang, Xiufang Gong, Lina Hao, Min Cao, Dmitry A. Rodionov, Changjun Wang, Youjun Feng

**Affiliations:** ^1^ Department of Medical Microbiology and ParasitologyZhejiang University School of MedicineHangzhouZhejiang310058China; ^2^ A.A. Kharkevich Institute for Information Transmission ProblemsRussian Academy of SciencesMoscow127994Russia; ^3^ Department of EpidemiologyResearch Institute for Medicine of Nanjing CommandNanjing210002China; ^4^ Luxembourg Centre for Systems BiomedicineUniversity of LuxembourgEsch‐sur‐AlzetteL‐4360Luxembourg

**Keywords:** AgaR, amino sugars, d‐galactosamine, *N*‐acetyl‐d‐galactosamine, *Streptococcus suis*, virulence.

## Abstract

Bacterial pathogens can exploit metabolic pathways to facilitate their successful infection cycles, but little is known about roles of d‐galactosamine (GalN)/*N*‐acetyl‐d‐galactosamine (GalNAc) catabolism pathway in bacterial pathogenesis. Here, we report the genomic reconstruction of GalN/GalNAc utilization pathway in *Streptococci* and the diversified *aga* regulons. We delineated two new paralogous AgaR regulators for the GalN/GalNAc catabolism pathway. The electrophoretic mobility shift assays experiment demonstrated that AgaR2 (AgaR1) binds the predicted palindromes, and the combined in vivo data from reverse transcription quantitative polymerase chain reaction and RNA‐seq suggested that AgaR2 (not AgaR1) can effectively repress the transcription of the target genes. Removal of *agaR*2 (not *agaR*1) from *Streptococcus suis* 05ZYH33 augments significantly the abilities of both adherence to Hep‐2 cells and anti‐phagocytosis against RAW264.7 macrophage. As anticipated, the dysfunction in AgaR2‐mediated regulation of *S. suis* impairs its pathogenicity in experimental models of both mice and piglets. Our finding discovered two novel regulators specific for GalN/GalNAc catabolism and assigned them distinct roles into bacterial infections. To the best of our knowledge, it might represent a first paradigm that links the GalN/GalNAc catabolism pathway to bacterial pathogenesis.

## Introduction

Amino sugars are referred to a variety of diversified/complex monosaccharides in which a hydroxyl group is chemically replaced with the amine group. Most of current knowledge on the metabolism of amino sugars comes from studies with *Escherichia coli* (Reizer et al. 1996), *Bacillus subtilis* (Freymond et al. 2006; Gaugue et al. 2014; Plumbridge 2015) and *Streptomycetes coelicolor* (Rigali et al. 2008). Relative to the best‐known examples of amino sugars, glucosamine (GlcN) and *N*‐acetylglucosamine (GlcNAc), the investigations on the other two amino sugar derivatives of galactose, *N*‐acetyl‐d‐galactosamine (GalNAc) and d‐galactosamine (GalN), are relatively limited, but increasingly accumulated (Abu‐Qarn et al. 2008; Leyn et al. 2012). It seemed very likely that GalN/GalNAc amino sugars as common components participate in the formation of various cell structures/constitutes in three domains of life (Abu‐Qarn et al. 2008; Plumbridge 2015). In general, not only does GalNAc act as an element of lipopolysaccharide displayed on bacterial cell wall (Bernatchez et al. 2005; Leyn et al. 2012), and but also connects carbohydrate chains in mammalian mucins (Carraway and Hull 1991). Additionally, it functions as the substrate in *N*‐acetyl *β*‐galactosidation, a new type of post‐translational modification of protein in organisms, including bacterial pathogens (Sadler et al. 1979; Barr and Nordin 1980; Davis et al. 1986; Abu‐Qarn et al. 2008). Given the multiple roles played by GalN/GalNAc, we therefore anticipated a hypothesis that GalN/GalNAc metabolism might be linked to bacterial infectivity.

**Figure 1:**
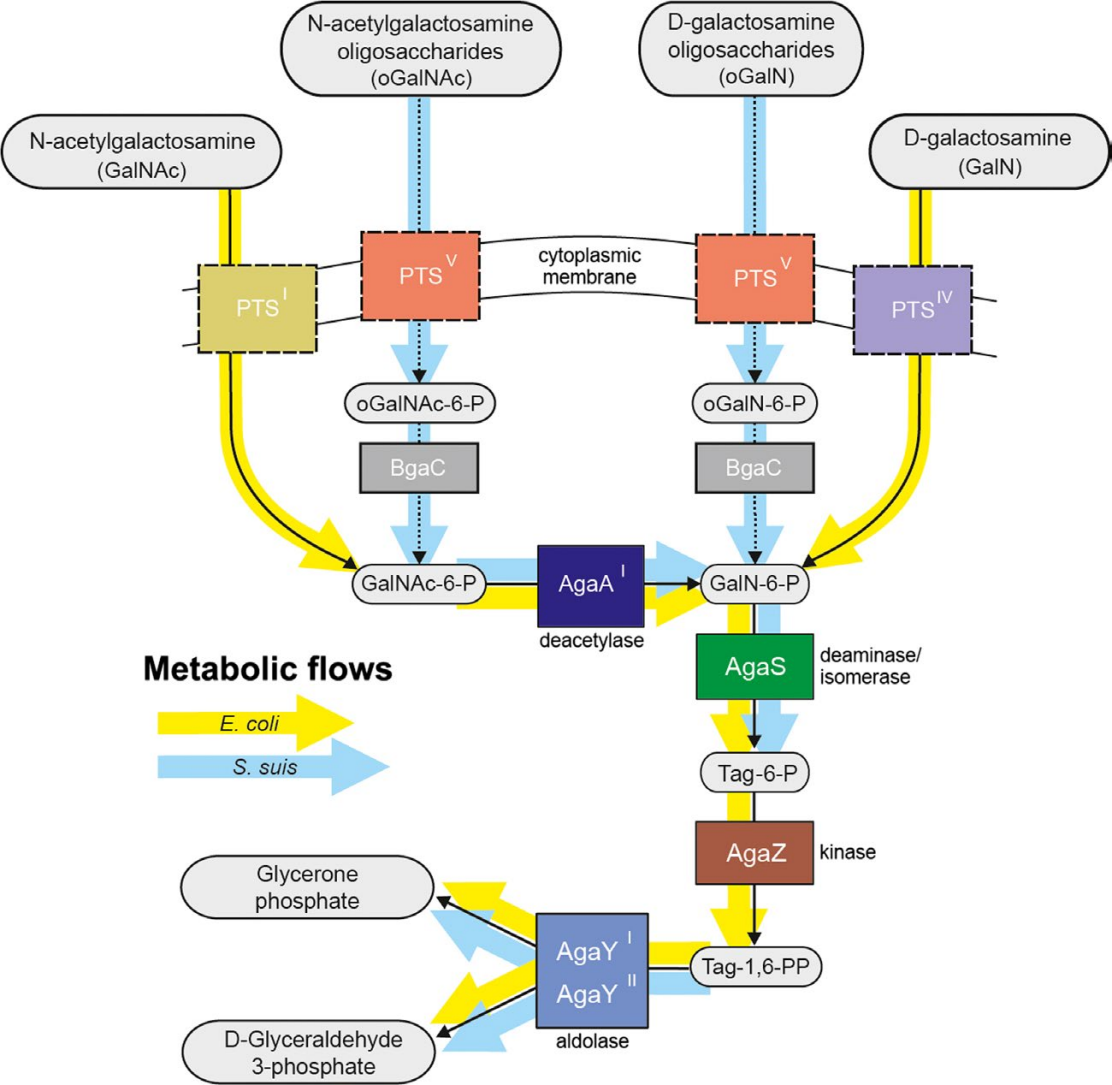
Reconstruction for GalNAc/GalN utilization pathways in *Streptococcus suis*. The variants of GalNAc/GalN pathway in *Escherichia coli* and *S. suis* are highlighted with yellow and light‐blue background arrows, respectively. GalNAc, *N*‐acetyl‐d‐galactosamine; GalN, galactosamine; PTS, phosphotransferase system; BgaC, *β*‐galactosidase; AgaA, GalNAc‐6‐P deacetylase; AgaS, GalN‐6‐P deaminase/isomerase; Tag, Tagtose; AgaZ, Tag‐6‐P kinase; AgaY, Tag‐1,6‐PP aldolase.

The paradigm pathway for GalN/GalNAc utilization/catabolism, which was initially proposed for *E. coli* in 1996 (Reizer et al. 1996), contained the following five steps (Fig. 1): (1) the transport and phosphorylation of GalN/GalNAc substrates (catalyzed by phosphortransferase system [PTS] systems AgaBCD and AgaVWEF, respectively) (Brinkkotter et al. 2000), (2) the AgaA‐mediated deacetylation of GalNAc‐6‐P (Reizer et al. 1996), (3) the deamination/isomerization of GalN‐6‐P by the bi‐functional enzyme AgaS into Tag‐6‐P (Reizer et al. 1996), (4) the AgaZ kinase‐aided phosphorylation of Tag‐1,6‐P from Tag‐6‐P (Reizer et al. 1996), and (5) cleavage of Tag‐1,6‐PP by the class II aldolase, AgaY (Brinkkotter et al. 2002), to produce glyceraldehyde 3‐phospahte and glycerone phosphate (PEP). Interestingly, a recent comparative genomics‐based study suggested an extensive diversity in GalN/GalNAc utilization pathways of Proteobacteria such as *Shewanella* (Leyn et al. 2012). In particular note the first two steps of this pathway exhibit of high variability, while the latter three steps are largely conserved (Leyn et al. 2012). In *E. coli*, not only does AgaR regulator that belongs to the DeoR family of transcriptional factors, act as an autoregulator, but also negatively controls the expression of two *aga* genes (*agaZ* and *agaS*) of GalN/GalNAc catabolism pathway via direct binding of the specific palindromes in front of these target genes (Ray and Larson 2004). Although the fact that both GalN and GalNAc can induce activities of these *aga* genes‐encoding protein products is aware, the physiological ligands for AgaR repressor remain unclear (Leyn et al. 2012).

*Streptococcus suis*, a Gram‐positive bacterium, is a zoonotic agent with the ability to infect both its natural host swine and human individuals with close contact with swine/pork‐related products (Feng et al. 2010). According to the differentiation in their bacterial capsule structure, this *Streptococcus* species is categorized into 35 serotypes (Feng et al. 2010, 2014b). Among them, serotype 2 (SS2) is generally believed to be most virulent, in that it is frequently isolated from clinical diseased swine (Ma et al. 2009; Feng et al. 2014b) and human sporadic cases (Feng et al. 2009b) and/or big‐scale outbreaks (Tang et al. 2006; Chen et al. 2007). We are aware that SS2 has spread to more than 30 countries/regions and claims nearly 1600 cases of human infections worldwide (Feng et al. 2014b). The molecular mechanism underlying bacterial pathogenicity has been partially delineated thus far (Feng et al. 2014b), and these identified virulence determinants include the previously‐known virulence factors exemplified with capsule (Benga et al. 2008; Seitz et al. 2014) and suilysin (Jacobs et al. 1996; Lun et al. 2003; Takeuchi et al. 2014). In addition to the regulatory networks amongst the newly identified factors (e.g., the two‐component systems SalK/SalR [Li et al. 2008], NisK/NisR [Xu et al. 2014], CiaRH [Li et al. 2011], etc.), it would be of particular interest to note the contributions of enzymes from central metabolisms (enolase [Eno] [Esgleas et al. 2008; Feng et al. 2009a; Zhang et al. 2009a; Lu et al. 2012], glutamine synthase [GlnA] [Si et al. 2009], Inosine 5‐monophosphate dehydrogenase [Impdh] [Zhang et al. 2009b; Zhou et al. 2014], etc.) to bacterial pathogenesis (Feng et al. 2014b). However, nothing is aware regarding the potential relevance of bacterial GalN/GalNAc metabolism and/or its regulation to *S. suis* infection.

In this work, we employed the integrative approaches combining the bioinformatics and comparative genomics to conduct the genomic reconstruction of the GalN/GalNAc utilization pathway in *Lactobacillaceae, including* the zoonotic pathogen *S. suis*. Also, we are the first to report the functional definition of two new GntR‐type transcription factors (referred to AgaR2 and/or AgaR1) with involvement of GalN/GalNAc catabolism. More intriguingly, we observed that AgaR2‐dependent regulation of GalN/GalNAc utilization pathway is required for bacterial virulence of *S. suis* serotype 2. To the best of our knowledge, it represents the first example that the genetic control of GalN/GalNAc catabolism is linked to bacterial infectivity of *Streptococcus*.

## Materials and Methods

### Bacterial strains, cell lines and growth conditions

Bacterial strains used here included derivatives of either *E. coli* or *S. suis* 2 (Table S1). *Escherichia coli* Topo10 and BL21 (DE3) are applied for gene cloning, and protein expression, respectively. The growth medium for *E. coli* and *S. suis* 2 is separately Luria‐Bertani (LB) broth and Todd‐Hewitt broth (THB; Difco Laboratories, Detroit, MI). These bacteria are grown at 37°C overnight. Given the selective pressure to ensure the replication of recombinant plasmids or the maintenance of engineered strains, appropriate antibiotics (Sigma, St. Louis, MO) were supplemented as follows: 100 *μ*g/mL of Spectinomycin for *S. suis*; either 100 *μ*g/mL of Ampicillin or 50 *μ*g/mL of Kanamycin for *E. coli*. The two kinds of cell lines used here corresponded to the human laryngeal epithelial cell Hep‐2 (CCTCC GDC004) and the mouse macrophagocyte Raw 264.7 (ATCC TIB‐71, Rockville, MD), respectively (Table S1) (Hu et al. 2014), and cultivated at 37°C in the presence of 5% CO_2_ in Dulbecco's modified Eagle's medium with 10% fetal bovine serum (Roche, Indianapolis, IN, USA),100 *μ*g/mL gentamycin, and 5 *μ*g/mL penicillin G (Feng et al. 2012).

### Plasmids and DNA manipulations

The *S. suis agaR*2 (*SSU05_0447*) gene was amplified using polymerase chain reaction (PCR) with primers *SSU05_0447*‐F plus *SSU05_0447*‐R (Table S2) and ligated into the BamHI and XhoI sites of pET28a(+) expression vector (Feng et al. 2008), resulting in the recombinant plasmid pET28*‐447* (Table S1). Similarly, the other expression plasmid pET28*‐448* was given through direct cloning of *S. suis agaR*1 (*SSU05_0448*) gene carrying BamHI and SalI sites introduced by primers (*SSU05_0448*‐F plus *SSU05_0448*‐R) into the expression vector pET28a(+) with the same cuts (Tables S1, S2). The above two plasmids (pET28*‐447* and pET28*‐448*) are designed to prepare in vitro proteins of AgaR2 and AgaR1, respectively. For functional complementation, the two genes (*SSU05_0447* and *SSU05_0448*) were separately inserted into the low‐copy shuttle vector pVA838 (Romero et al. 1987), giving the plasmids pVA838‐447 and pVA838‐448, respectively (Table S2). All the recombinant plasmids involved in this study were confirmed with both PCR assays and direct DNA sequencing.

### Expression and purification of two AgaR proteins

The two recombinant plasmids (pET28a‐*448* and pET28a‐*447*) were separately transformed into BL21 (DE3), giving the engineered strains FYJ356, and FYJ536, respectively (Table S1). The two versions of hexahistidine‐tagged AgaR protein (referred to AgaR1 [SSU05_0448] and AgaR2 [SSU05_0447]) were produced using the above two engineered strains FYJ356 and FYJ536, respectively. In brief, when bacterial optical density at wave‐length of 600 nm (OD600) reached 0.6–1.0, the bacterial cultures with an appropriate plasmid (Table S1) were induced with 0.5 mmol/L isopropyl‐*β*‐d‐thiogalactopyranoside (IPTG) at 30°C for 3–5 h. As we described before (Feng and Cronan 2009), two rounds of French pressure‐based lysis were conducted for release of the recombinant protein AgaR1 (and/or AgaR2), and subsequent procedures of protein purification included the nickel column‐based affinity purification followed by fast phase liquid chromatography (FPLC) (Feng et al. 2008; Feng and Cronan 2012). Consequently, the protein of interest was concentrated via ultrafiltration (Feng et al. 2008), and the purity was judged by 12% sodiumdodecyl sulfate polyacrylamide gel electrophoresis (SDS‐PAGE).

### Western blotting

Western blot was performed routinely to further verify the hexahistidine‐tagged AgaR1 (and/or AgaR2) protein. The protein samples were separated with 12% SDS‐PAGE and thereafter transferred to a nitrocellulose membrane (Amersham, GE Healthcare, Piscataway, NJ, USA). The primary antibody was an anti‐hexahistidine mouse monoclonal antibody, and the secondary antibody was a peroxidase‐conjugated goat anti‐mouse immunoglobulin G. The signal of target protein band was captured by an exposure to the high‐performance chemiluminescence ECL film (Amersham, GE Healthcare, Piscataway, NJ, USA).

### Liquid chromatography quadrupole time‐of‐flight mass spectrometry

To verify the identity of the recombinant AgaR1 (and/or AgaR2), the resultant peptides by digestion with Sequencing Grade Trypsin (G‐Biosciences, St. Louis, MO, 12.5 ng *μ*L^−1^ in 25 mmol/L ammonium bicarbonate) were subjected for analyses of A Waters Q‐Tof API‐US Quad‐ToF mass spectrometer linked to a Waters nano Acquity UPLC (Feng et al. 2013b). The mass data acquired were assayed through Waters Protein Lynx Global Server 2.2.5, Mascot (Matrix Science, Boston, MA, USA) combined with BLAST against NCBI nr database (Feng et al. 2014a).

### Size exclusion chromatography and chemical cross‐linking assays

The nickel column‐based purified AgaR1 (and/or AgaR2) protein was further assayed by gel filtration chromatography using a Superdex 75 column (Amersham, GE Healthcare, Piscataway, NJ, USA) run on an Äkta fast protein liquid chromatography system (GE Healthcare) as described previously (Feng and Cronan 2010, 2011). The column effluent was evaluated at a flow rate of 0.5 mL/min in PBS buffer (10 mmol/L Na_2_HPO_4_, 2 mmol/L KH_2_PO_4_, 20 mmol/L Tris‐HCl, 137 mmol/L NaCl, 2.7 mmol/L KCl, pH 7.4). The protein peak at the position of expected elution volume was sampled and verified by 12% SDS‐PAGE.

To further elucidate the solution structure of AgaR1 (and/or AgaR2) protein from *S. suis*, we conducted chemical cross‐linking experiments in which ethylene glycol bis‐succinimidylsuccinate (EGS) (Pierce, Rockford, IL, USA) was added (Feng et al. 2008). In each reaction system (15 *μ*L in total), the interested protein (~5 mg/mL) was separately incubated with the EGS cross‐linker (at different levels [0.1, 0.2, 0.5, 1.0, 2.5, 5, 10, 20 *μ*mol/L for AgaR1 protein; 0, 2.5, 5, 10, 20 *μ*mol/L, for AgaR2 protein]) for 1 h at room temperature. Finally, the reaction products were visualized via 12% SDS‐PAGE followed by commassiee brilliant blue staining.

### Electrophoretic mobility shift assays

The function of the predicted AgaR2 and AgaR1 binding sites were proved using electrophoretic mobility shift assays (EMSA) as we established earlier (Feng and Cronan 2011; Feng et al. 2013a,b) with minor modifications. All the double‐strand DNA probes were produced in vitro by annealing two complementary oligonucleotides in TEN buffer (10 mmol/L Tris‐HCl, 1 mmol/L Ethylene Diamine Tetraacetic Acid (EDTA), 100 mmol/L NaCl; pH 8.0), and labeled with DIG‐ddUTP (Roche, Indianapolis, IN, USA) by the terminal transferase (Feng and Cronan 2011, 2012). Three AgaR1‐specific DNA probes used here included *SSU05_0448/9* site1 probe (38 bp), *EF814* site1 probe (38 bp), and *EF1809* site1 probe (38 bp). In addition to *SSU05_0447* probe (36 bp) as the negative control, the other four tested AgaR2‐recognizable sites corresponded to *SSU05_0195* probe (36 bp), *SSU05_1259* probe (36 bp), 5 *SSU05_0448/9* site2 (36 bp), and *EF1809* site2 probe (36 bp) (Table S2). After 20 min of incubation of the DIG‐labeled DNA probes (0.2 pmol) with or without AgaR2 (and/or AgaR1) protein in the binding buffer (Roche) at room temperature, the DNA–protein complexes were separated by the native 7% PAGE gel and transferred onto an equilibrated, positively charged nylon membrane (Roche) by contact blotting followed by UV cross‐linking (120 mJ for 180 sec) (Feng and Cronan 2011; Feng et al. 2013b). Finally, the signals were captured by exposure to the high‐performance chemiluminescence film (Amersham Hyperfilm ECL) (Feng and Cronan 2009, 2010).

### RNA isolation and real‐time qPCR

Mid‐log phase cultures of *S. suis* 2 strains (wild type [WT], Δ*agaR*1, Δ*agaR*2, CΔ*agaR*1 and CΔ*agaR*2) grown in THB media (with/without GalNAc‐6P) were collected to prepare the total bacterial RNA using an RNeasy bacterial RNA isolation kit (Qiagen, Hilden, Germany) (Feng et al. 2008; Li et al. 2008). As we performed before (Feng and Cronan 2009, 2010), RNA integrity/quality was validated by separation of 1.0% agarose gel electrophoresis. The possible contamination of trace genomic DNA in the RNA samples was ruled out by PCR‐based detections, using the total RNA as the template (Feng and Cronan 2009, 2010, 2011).

Using the qualified RNA preparations, complementary DNAs (cDNAs) were synthesized by reverse transcription (RT). Then, the real‐time quantitative PCR (qPCR) combined with the SYBR green method (Feng et al. 2008; Feng and Cronan 2009) was carried out to probe possible relevance of *agaR*2 (and/or *agaR*1) to the altered expression profile of genes encoding GalNAc utilization pathway. The method of 2^−ΔΔ*CT*^ (34) was applied to determine the relative level of the target genes associated with GalNAc utilization in which the 16S rRNA‐encoding gene *16S rDNA* as the internal reference (Table S2). Target genes tested here are *agaS* (*SSU05_0195*), *agaAY* (*SSU05_1258/9*), *bgaC* (*SSU05_0449*), and *gadVWEF* (*SSU05_0450*,* SSU05_0451*,* SSU05_0452* and *SSU05_0453*), respectively.

### Construction of Δ*agaR*2 (and Δ*agaR*1) mutants and functional complementation

The *agaR2* (or *agaR*1) gene from the *S. suis* 2 strain 05ZYH33 was replaced with the spectinomycin resistance (Spc^*R*^) cassette by homologous recombination (Feng et al. 2008, 2012; Hu et al. 2014). Briefly, the Spc^*R*^ cassette from pSET2 (Takamatsu et al. 2001) was cloned into the pUC19 vector (Invitrogen) to give the intermediate plasmid pUC19‐Spc (Table S1), and then the two DNA fragments adjacent to the *agaR2* (or *agaR1*) gene were separately inserted into the pUC19‐Spc vector, giving the knockout plasmid pUC*::447* and pUC*::448*, respectively (Table S1). As we did before (Feng et al. 2008; Li et al. 2008), the plasmid of pUC*::447* (or pUC*::448*) was electroporated into the competent cells of *S. suis* 05ZYH33 to acquire positive transformants with Spc^*R*^. The multiplex‐PCR techniques were adopted to screen the Δ*agaR*2 (and/or Δ*agaR*1) mutant (Table S2). Consequently, the mutants we acquired for functional experiments were further proved by direct DNA sequencing. The two plasmids of pVA838*‐447* and pVA838*‐448* were separately transformed into Δ*agaR*2 and Δ*agaR*1 mutants to give the complemented strains CΔ*agaR*2 and CΔ*agaR*1*,* respectively (Table S2).

### Assays for ability of bacterial adherence and phagocytosis

As Hytönen et al. (Hytonen et al. 2006) reported, *S. suis* bacteria (WT, Δ*agaR*2 and CΔ*agaR*2) grown in the mid‐log phase were subjected to cell lines‐based analyses. The two cell lines are Hep‐2 (human laryngeal epithelial cell line) and murine macrophage Raw 264.7 cells (Feng et al. 2012; Hu et al. 2014). Bacterial adherence was tested with Hep‐2 cell line, and the evaluation for ability of anti‐phagocytosis was based on the Raw264.7 cells.

### Infection assays of experimental animals

To reveal the role of *agaR*2 (and/or *agaR*1) in bacterial pathogenesis/virulence, two different kinds of experimental animals were employed, including BALB/c (4‐week old, female) mice and SPF‐piglets. In the infection experiment of mice, totally 60 animals were challenged that are classified into six groups (10 mice/group). Except that THB acted as a negative control, the other five groups infected with *S. suis* 2 (at a dose of 1 × 10^8 ^CFU per mouse) corresponded to WT, Δ*agaR*2, CΔ*agaR*2, Δ*agaR*1, and CΔ*agaR*1, respectively.

The result obtained from the experiment of mice infection was further checked using the infection test of piglets, its natural host of *S*. *suis*. Given the fact that only *agaR*2 (not *agaR*1) plays a role in bacterial infectivity, three groups of piglets (six piglets/group) were rechallenged by WT, Δ*agaR*2, and CΔ*agaR*2, respectively. Clinical syndromes of the infected mice/piglets were monitored for 72 h. Of particular note, deaths were recorded and moribund animals were humanely killed. All experiments on live vertebrates in this study were approved by the Ethics Committee of Research Institute for Medicine of Nanjing Command and performed in accordance with the relevant guidelines and regulations (Cao et al. 2011; Feng et al. 2012; Hu et al. 2014).

### Bioinformatics analyses

Genome sequences were downloaded from the MicrobesOnline genomic data base (Dehal et al. 2010). Identification of orthologs was performed using the BLASTP search in the non‐redundant database (Altschul et al. 1997) and MicrobesOnline tree browser. For functional protein annotations by distant homology to characterize proteins, BLAST search in the SwissProt/UniProt database was used. Analysis of chromosomal gene clustering was performed by MicrobesOnline and SEED web resources (Dehal et al. 2010; Disz et al. 2010). The GalNAc utilization subsystem curation and analysis were conducted, using the SEED platform (Disz et al. 2010). Protein domains were determined by protein similarity search tools in the Pfam database (Sonnhammer et al. 1998). Multiple sequence alignments were constructed by either ClustalW (http://www.ebi.ac.uk/Tools/clustalw2/index.html) (Thompson et al. 2002) or MUSCLE (Edgar 2004). Phylogenetic trees were constructed using the maximum likelihood algorithm implemented in the PHYLIP package (Felsenstein 1996) and visualized via the dendroscope tool (Huson et al. 2007). Sequences Logos were constructed using WebLogo package (Crooks et al. 2004).

For genomic reconstruction of the regulons, we used the well‐established comparative genomics approach (Rodionov 2007). The approach includes inference of transcriptional factor‐binding sites (TFBSs), construction of nucleotide positional weight matrices (PWMs) for TFBSs motifs, and reconstruction of regulons in complete genomes on the basis of prediction of putative TFBSs in the promoter gene regions. First, in the studied *Lactobacillales* genomes, we revealed orthologs of previously known genes for GalNAc utilization (Table S3). Second, we predicted possible transcriptional regulators for the uncovered GalNAc utilization. Candidate regulators were attributed to the regulons by using a genomic colocalization and co‐occurence of a putative regulator with the identified GalNAc utilization genes. Such analysis defined two groups of transcriptional regulators belonging to the HutC subfamily of the GntR family (Fig. 7). For each group of AgaR proteins, we identified putative binding motifs analyzing upstream regions of presumably regulated gene by the Discover Profile tool implemented in the RegPredict Web server (Novichkov et al. 2010). In this approach, putative TFBSs were determined as overrepresented words in upstream regions of putatively co‐regulated genes. Based on the genomic co‐occurrence and co‐presence of genes, it has been proposed that AgaR1‐binding sites should be located upstream hydrolase and PTS genes while AgaR2 sites ought to be upstream of deacetylase, isomerase, and aldolase genes. For the prediction of putative TFBSs, we analyzed upstream regions of probably related operons, expanding −400 to +100 bp relative to start codon of the first gene of the operon. Both AgaR1 and AgaR2 belong to HutC subfamily. TFBSs for HutC subfamily proteins have structure of an even palindrome (Rigali et al. 2002; Suvorova et al. 2015). Thus, for prediction of the AgaR1 and AgaR2 TFBSs motifs, we searched for even palindromic DNA motifs of 14–24 bp. Among all motifs found for each set of upstream regions, we selected the longest motif with the highest information content. The selected motif was quality controlled by two approaches, (1) consistency check and (2) phylogenetic footprinting. Consistency check (Mironov et al. 1999; Rodionov 2007) was done, that is, motif was checked for the presence in multiple number of genomes. High quality motif should find predicted TFBSs in upstream regions of predicted regulated operons in genomes having the analyzed regulator, but not in genomes lacking the regulator. Phylogenetic footprinting technique is based on analysis of multiple alignments for upstream regions of orthologous genes (Shelton et al. 1997). High quality motif should find predicted TFBSs that are located inside conserved islands of multiple alignments. When the high quality of the motif was confirmed by both consistency check and phylogenetic footprinting, the identified regulatory motifs were used for the construction of the PWMs (profiles) and the obtained matrices were used to determine additional candidate regulatory sites in the analyzed genomes, using the Run Profile tool in the RegPredict Web server. The scores of sites were calculates as a sum of nucleotide weights for each position.

### Statistics

The data used here were expressed as mean ± SD. Unless specified, data were analyzed by two‐tailed, unpaired *t* test, and all assays were repeated no less than three times. The threshold for significance refers to the *P* < 0.05.

## Results

### Reconstruction of the GalNAc utilization pathway in *Lactobacillaceae*

The GalNAc utilization pathway has been previously reconstructed by the comparative genomics approach in a large number of *Proteobacteria* species (Leyn et al. 2012) and experimentally analyzed in *E. coli* (Brinkkotter et al. 2000) and *Shewanella* sp. ANA‐3 (Leyn et al. 2012). Here we used the same approach for reconstruction of the GalNAc utilization pathway in the zoonotic agent, *S. suis* (Fig. 1 and Table S1). For integrated genomic reconstruction of GalNAc utilization pathways and concordant transcriptional regulation, we searched orthologs of known GalNAc utilization genes (Leyn et al. 2012) in complete *Lactobacillales* genomes. As a result, the genes encoding, this metabolic pathway was identified in 16 genomes representing four families, *Streptococcaceae*,* Lactobacillaceae*,* Enterobacteraceae*, and *Carnobacteriaceae* (Table S1). The minimal number of genes in the reconstructed regulons was detected in *Lactobacillus helveticus* and *Streptococcus pyogenes*. In these organisms, only one or two genes from the GalNAc utilization pathway were found because these genes were insufficient for GalNAc utilization. Most probably, these organisms cannot utilize GalNAc. The minimal gene set allowing utilization of GalNAc is present in a group of closely related genomes including *Streptococcus gordonii*,* Streptococcus mitis*, and *Streptococcus pneumonia*. This gene set contains genes for regulator (*agaR*), galactosamine‐6‐phosphate deaminase/isomerase (*agaS*), glycoside hydrolase (*bgaC*), and PTS (*gadVWEF*). The other well‐studied *Streptococcus* genomes also contain genes for the tagatose‐1,6‐diphosphate aldolase (*agaY*). In contrast, all the analyzed *Lactobacillus* lack *agaY* gene but contain the gene for *N*‐acetylgalactosamine‐phosphate deacetylase (*agaA*). Amongst *Streptococcaceae*,* agaA* gene was identified only in the *S. suis* genome. The gene for tagatose‐6‐phosphate kinase (*agaZ*) was found only in *Enterococcus faecalis* and *Carnobacterium* sp. 17‐4.

Previously *agaY* gene was shown to be not obligatory for the GalNAc utilization and function of *agaZ* was proposed to be actualized by other genes, such as *lacC* (EC 2.7.1.144) or *pfk* (EC 2.7.1.11). Orthologs of these genes were found in the studied genomes, for example in *S. suis* LacC is encoded by gene with locus tag *SSU05_1041* and Pfk is encoded by *SSU05_0543*.

The presence or absence of *agaA* gene is the crucial point for the ability or disability to utilize GalNAc. Thus, the absence of this gene in the Aga regulons points to the ability of the organism to utilize only GalN, but not GalNAc utilization. Thus, organisms lacking *agaA* gene should have GalN‐specific transporters whereas organism having *agaA* gene should be able to transport GalNAc (Leyn et al. 2012). To predict the specificity of the *Lactobacillaceae* transport systems, we compared all the identified PTSs to the previously described ones. The phylogenetic analysis revealed that all the *Lactobacillaceae* PTSs are orthologous to the transport system from *Haemophilus parasuis* (Fig. S4). This PTS was previously proposed to be specific to GalN‐containing oligosaccharides. Co‐occurrence and co‐localization of this PTS with the hydrolase genes in the most studied genomes confirms its specificity to the oligosaccharides. On the other hand, PTS genes are co‐localized also with the *agaA* gene in the large number of studied genomes (Table S3) that designate to the possibility of this system to transport GalNAc or both GalNAc and GalN containing oligomers. However, we cannot have success in predicting the precise specificity of the *gadVWEF* encoded PTS, using only the comparative genomics approach.

### Comparative genomics‐based insights into the regulation of GalNAc utilization in *Lactobacillales*

**Figure 2:**
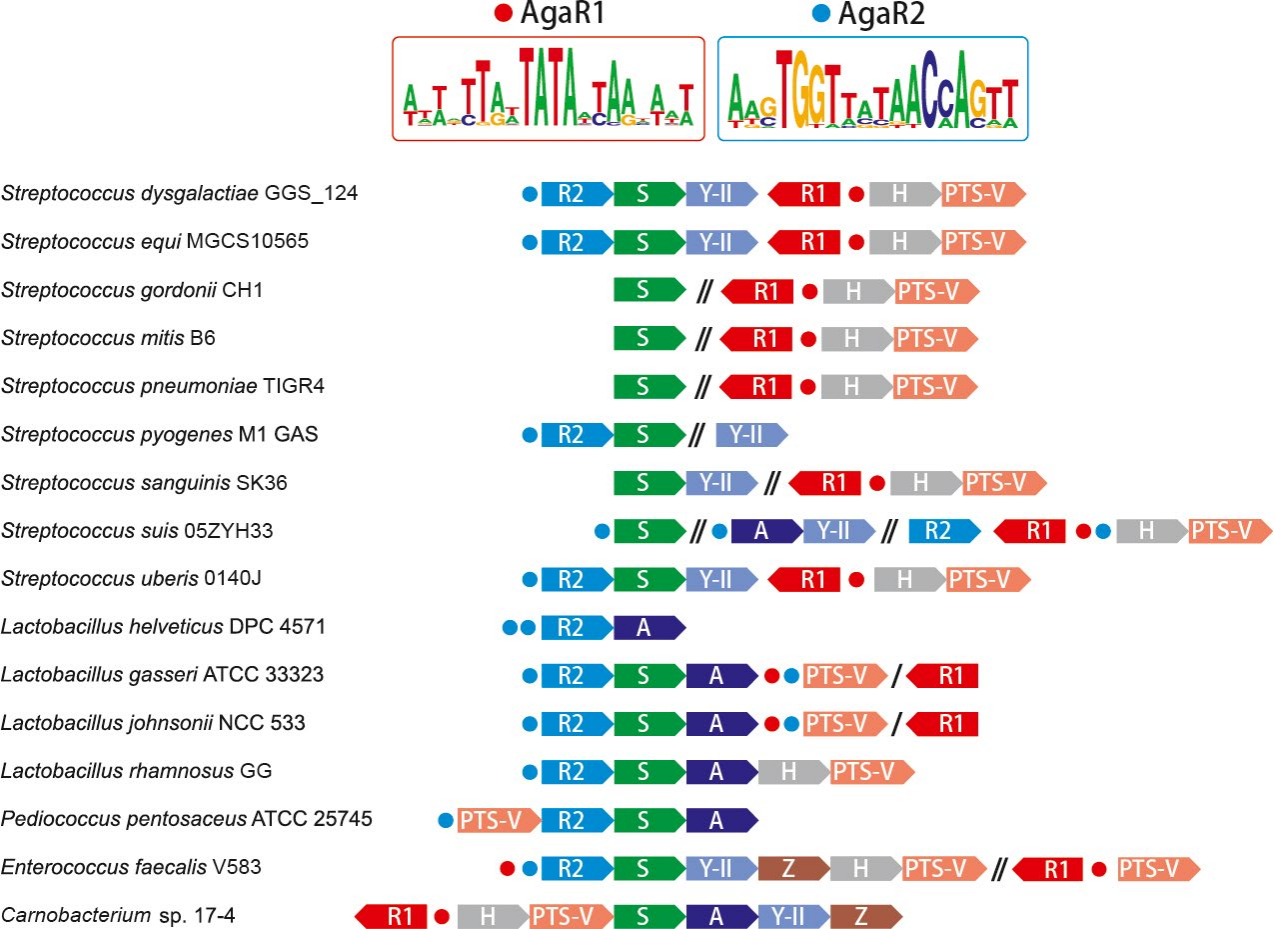
Discovery of the genome‐wide regulons encoding the GalNAc/GalN utilization pathways in *Lactobacillales* Arrows represent the GalNAc/GalN catabolism‐related genes, and circles denote the predicted AgaR‐recognizable sites. The genes are variably colored according to differential functions assigned in Figure 1 and labeled with the last letter of the corresponding protein. Genes from the same genetic loci that are not adjacent/neighbored each other are separated by a slash. Similarly, genes from different genetic loci are separated by a double slash. Given the two types of putative AgaR‐binding palindromes shown on the top (details in Table S4), AgaR1 and AgaR2 sites are accordingly colored with red and blue. Sequence logos were given using the WebLogo package (http://weblogo.berkeley.edu/logo.cgi). The detailed information on the displayed loci is listed in Table S3 and Figure S4. GalNAc, *N*‐acetyl‐d‐galactosamine; GalN, galactosamine.

**Figure 3:**
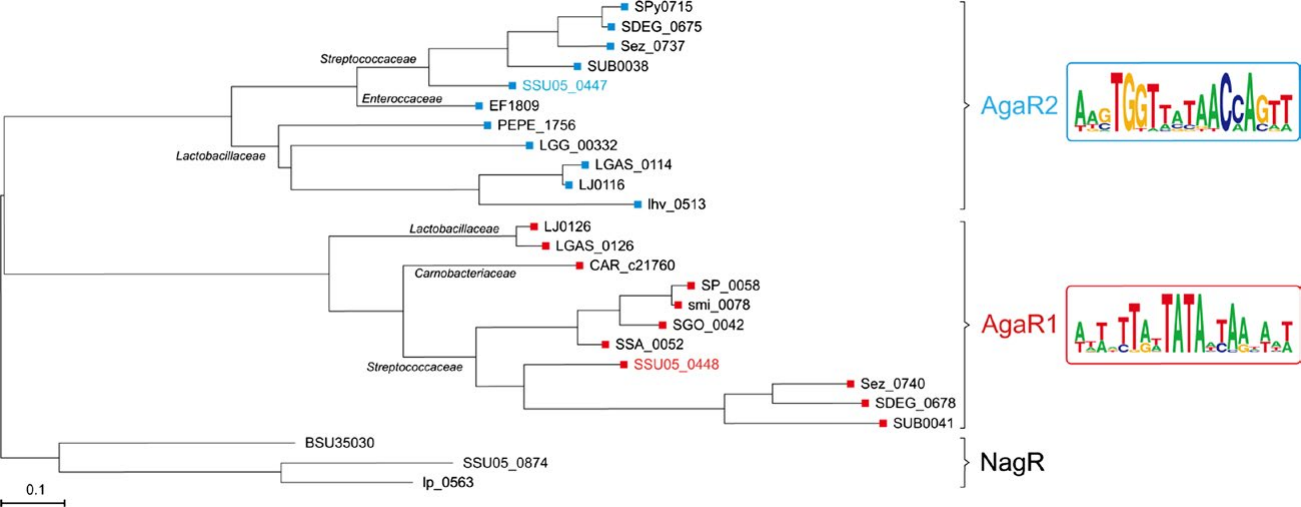
Maximum‐likelihood phylogenetic tree of AgaR proteins. AgaR1 (SSU05_0448) is given in red, and AgaR2 (SSU05_0447) is highlighted in blue. The NagR proteins from *Bacillus subtilis*,* Streptococcus suis*, and *Lactobacillus plantarum* are used as outtree. The logos for the predicted palindromes recognized by AgaR1 (AgaR2) is presented on the right hand. All the homologs of AgaR1 (AgaR2) is accessed to Table S3. NagR proteins from *B. subtilis* 168 (Bertram et al. 2011), *L. plantarum* WCFS1, and *S. suis* 05ZYH33 were used as an outgroup. NagR was selected because, as like AgaR1 and AgaR2, this protein is a member of the HutC subfamily of the GntR family and a regulator of aminosugar metabolism.

For the integrated genomic reconstruction of GalNAc utilization pathways and the concordant transcriptional regulation, we searched orthologs of known GalNAc utilization genes (Leyn et al. 2012) in complete *Lactobacillales* genomes. As a result, the genes encoding this metabolic pathway were identified in 16 genomes representing four families, *Streptococcaceae*,* Lactobacillaceae*,* Enterobacteraceae*, and *Carnobacteriaceae* (Table S3). Analysis of conserved chromosomal loci with the GalNAc utilization genes detected the presence of one or two suggested transcriptional regulators per genome (Fig. 2 and Table S3). All predicted regulators belong to the GntR family of transcription factors (Hoskisson and Rigali 2009). Phylogenetic analysis revealed that the predicted regulators form two separate orthologous groups that we named AgaR1 and AgaR2 (Fig. 3). A strong tendency of *agaR*1 and *agaR2* genes to cluster onto the chromosome with the GalNAc utilization genes suggests conservation of their function (Fig. 2). Two *Lactobacillaceae* and four *Streptococcaceae* genomes have both *agaR*1 and *agaR2* genes, whereas in other genomes only one of the regulator genes was present (Fig. 2 and Table S3).

To infer the AgaR1/AgaR2 regulons in *Lactobacillales*, we used the comparative genomics approach applied in the RegPredict Web server. This approach combines prediction of candidate regulator‐binding sites with cross‐genomics comparison of regulons. Since AgaR1 and AgaR2 protein orthologous groups are distantly related to each other (30% identity), we propose that their binding motifs should also be different. In most analyzed genomes, *agaR*1 is co‐localized with genes for glycoside hydrolase and PTS, whereas *agaR2* tends to be clustered onto the chromosome with the genes for deacetylase, isomerase, and aldolase (Fig. 2 and Table S3). Thus, we proposed that AgaR1‐binding sites should be located upstream hydrolase and PTS genes while AgaR2 sites ought to be upstream of deacetylase, isomerase, and aldolase genes. Upstream regions of genes presumably regulated by each transcriptional factor were analyzed using Discover Profile tool of the RegPredict Web resource. After the identification of putative‐binding motif, we searched for additional regulatory sites in the analyzed genomes and finally reconstructed AgaR1 and AgaR2 regulons.

The candidate motifs for both the studied regulators have an even palindrome structure (Figs. 2, 5A and Table S4) that is in good agreement with previous observations on binding motifs for proteins of the HutC subfamily of GntR family (Hoskisson and Rigali 2009). Predicted AgaR1‐binding sites have a length 20 bp, whereas the predicted‐binding sites for AgaR2 are 18 bp sequences (Fig. 5A and Table S4). Both AgaR1 and AgaR2‐binding motifs have a palindrome AT‐rich central part. The AgaR1‐binding motif is AT‐rich at all times and quite degenerated, that is, has moderate information content. On the other hand, the AgaR2‐binding motif is more CG‐rich and conserved and has a higher information content than AgaR1‐binding motif. Additionally, AgaR2‐binding motif demonstrate similarities with the motifs of some regulators, previously characterized experimentally or in silico, such as GnbR of *Lactobacillus casei* BL23 (Bidart et al. 2014) and NagR proteins of *B. subtilis* (Bertram et al. 2011; Leyn et al. 2013) and various *Lactoacillales* (Ravcheev et al. 2013). Composition of the AgaR1 and AgaR2 regulons varies between species (Fig. 2). In *Lactobacillus rhamnosus (L. rhamnosus)*,* Enterococcus faecalis (E. faecalis)*, and *Carnobacterium* sp. 17‐4, all genes for GalNAc utilization are organized in a single operon that is regulated by a single regulator, AgaR2 in the first two genomes and AgaR1 in the last one (Fig. 2 and Table S3). In the most part of the *Streptococcaceae* and *Lactobaciilaceae* genomes, we observed a strong “division of labor” between the two regulators (Fig. 2). Thus, AgaR1‐binding sites were found upstream of hydrolase/PTS genes, whereas AgaR2 looks to regulate genes for deacetylase/isomerase/aldolase (Fig. 2). In the genomes of *S. suis*,* Lactobacillus gasseri (L. gasseri)* and *Lactobacillus johnsonii (L. johnsonii)*, overlapping of regulons was detected (Fig. 2). In all these genomes, genes for the transport system seemed likely to be under double regulation by AgaR1 and AgaR2. Also, in the vast majority of the analyzed genomes autoregulation was identified for both *agaR*1 and *agaR2* genes (Table S4).

### Characterization of AgaR1 and AgaR2, two novel GntR‐type regulators

**Figure 4:**
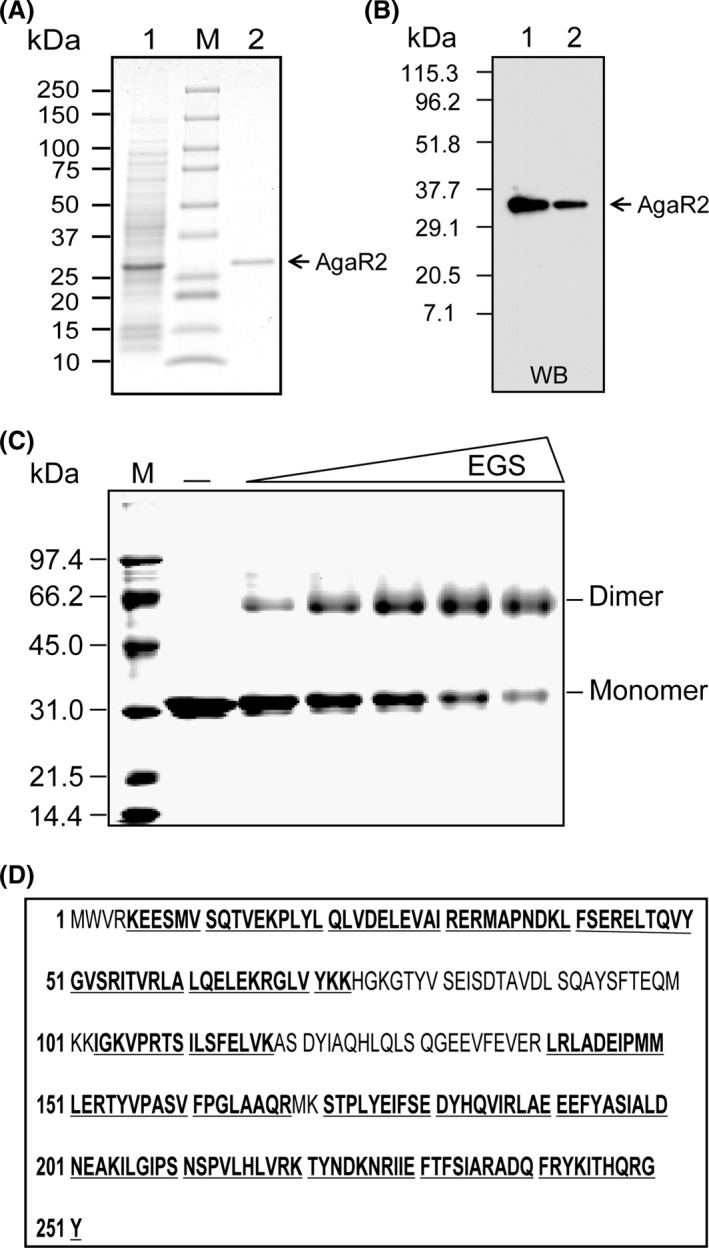
Preparation, identification, and characterization of the AgaR2 (SSU05_0447) protein. (A) 12% SDS‐PAGE profile of the purified AgaR2 (SSU05_0447) protein from *Streptococcus suis*. The protein with expected size (~31 kDa) is indicated with an arrow. “M” is the abbreviation for protein molecular weight. The numbers on left hand represent protein size (kDa). 1, crude extract of *Escherichia coli* lysate expressing the recombinant AgaR2 protein; 2, the purified form of the recombinant AgaR2 protein. (B) Western blotting analyses for the N‐terminal 6x his tagged AgaR2 protein using the anti‐6xHis tag primary antibody. WB, western blot; 1, the crude extract of *E. coli* lysate expressing the recombinant AgaR2 protein; 2, the purified form of AgaR2 protein. (C) Chemical cross‐linking assays for the solution structure of the AgaR2 protein. The chemical cross‐linker used here is EGS. The triangle on the top represents the addition of EGS cross‐linker in varied concentrations (0, 2.5, 5, 10, 20 *μ*mol/L in the right‐hand five lanes [left to right]). (M) Molecular weight. The molecular weight of the monomeric AgaR2 protein is estimated to be ~31 kDa, and the dimeric form is ~62 kDa. The protein sample was judged with 12% SDS‐PAGE. (D) MS‐based identification of the purified AgaR2 protein. The tryptic peptides with hits to the AgaR2 sequence are given in bold and underlined type. SDS‐PAGE, sodiumdodecyl sulfate polyacrylamide gel electrophoresis; EGS, ethylene glycol bis‐succinimidylsuccinate.

To probe the putative function of the two new members (*S. suis* AgaR2 and AgaR1) of the GntR family of transcription factors, we employed BL21(DE3)/pET28(a) a prokaryotic expression system to prepare the above two proteins in vitro. Consequently, the N‐terminal hexahistidine tagged *S. suis* AgaR2 protein was purified to homogeneity and gave a single‐protein band of appropriate molecular mass (~31 kDa for monomer) (Fig. 4A). The 6xHis tagged version of this recombinant *S. suis* AgaR2 protein was also determined by Western blot using the anti‐6xHis tag primary antibody (Fig. 4B). Chemical cross‐linking assays with the EGS cross‐linker visualized clearly the EGS dose‐dependent dimerization of AgaR2, indicating its predominant solution structure of this protein is a dimer (Fig. 4C). Liquid chromatography mass spectrometry‐based determination of tryptic peptides of the recombinant AgaR2 protein band excised from an SDS‐PAGE gel (Fig. 4A) verified its identity, in that the peptides matched *S. suis* SSU05_0447 protein with 77% coverage of the expected peptides (Fig. 4D).

Somehow different from the AgaR2 protein, two forms of AgaR1 protein (monomer, the predominant form, and trace amount of dimer) still can be detected by the separation with 12% SDS‐PAGE (Fig. S2A). Given that two possibilities are present (either the contaminated protein with the molecular mass at the dimeric position, or the dimeric form of AgaR1), Western blotting with the anti‐6xHis tag primary antibody was conducted. As anticipated, it clearly showed that two protein bands with an appropriate molecular mass (~31 kDa for monomer and ~60 kDa for dimer), ruling out the possibility of protein contamination (Fig. S2B). This observation is unexpected, but not without precedent. In fact we recently encountered a similar scenario in the case of the *Brucella* BioR regulator that is also a member of the GntR family transcription factor (Feng et al. 2013a). The essence of BioR1 forming a dimer was further proved by chemical cross‐linking assays (Fig. S2C). MS‐based analyses of two protein bands of AgaR1 (one is cut from a dimer, the other is collected from a monomer) validated exactly the identity wherein the two forms of peptides covered *S. suis* SSU05_0448 protein at the level of no less than 80% (Fig. S2D and E).

### *Streptococcus suis* AgaR2 (AgaR1) binds the predicted cognate palindromes

**Figure 5:**
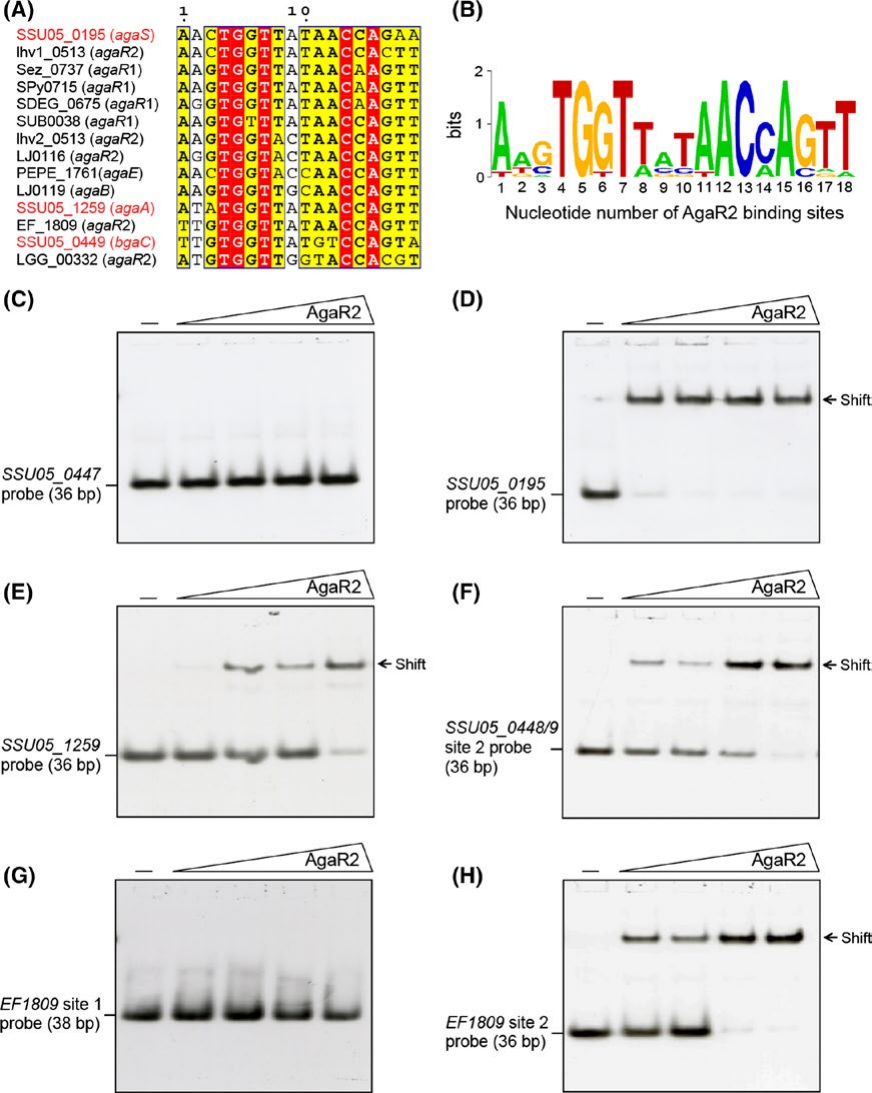
Binding of AgaR2 to the predicted cognate sites. Alignment of the AgaR2‐binding sites from species of *Lactobacillales* (A) and the resulting sequence logo (B). In (A) the identical residues are white letters in red background, similar residues are black letters in yellow background, and varied residues are in black letters. Names and locus tags for genes from *Streptococcus suis* are shown by red font. In (B) the sequence logo is generated using WebLogo (http://weblogo.berkeley.edu/logo.cgi). The detailed information of the cognate sites is seen in Table S4. (C) AgaR2 does not bind its own promoter. (D) Binding of *agaS* (*SSU05_0195*) promoter to AgaR2 proteinInteraction of AgaR2 protein with the promoter regions of both *agaA* (*SSU05_1259*) (in E) and *bgaC* (SSU05_0449) (in F) AgaR2 protein from *Streptococcus suis* cannot bind to the AgaR1 site of *EF1809* of *Enterococcus faecalis* V583 (in G), whereas it binds to its AgaR2 site (in H). The minus sign denotes no protein of AgaA2 added. The protein levels of AgaR2 (in the right hand four lanes of each panel [left to right]) were 0.5, 1, 2 and 5 pmol. The protein samples were incubated with 0.2 pmol of DIG‐labeled probe in a total volume of 20 *μ*L. A representative result from three independent gel shift assays (7% native PAGE) is given.

It seemed very likely that the genome of *S. suis* 05ZYH33 (Accession no.: CP000407.1) encodes a fully functional GalNAc utilization machinery and most of the genes encoding this pathway are regulated by AgaR2 (and/or AgaR1) (Fig. 1). Thereby, we employed EMSA to probe the binding of the AgaR2 (and/or AgaR1) protein to the cognate palindromes (Table S4, Fig. 5A and B). On the *S. suis* chromosome, totally three putative AgaR2‐binding sites (*SSU05_0195* probe, *SSU05_1259* probe and *SSU05_0448/9* site 2 probe) were localized, whereas only one possible AgaR1‐recognizable site (*SSU05_0448/9* site 1 probe) was detected. Because of the absence of the predicted AgaR2‐binding site upstream of the *agaR*2 gene, we expected that this gene is not autoregulated. Indeed, EMSA experiments suggested that AgaR2 protein cannot bind to its own promoter region (*SSU05_0447* probe), ruling out the possibility of autoregulation by AgaR2 regulator (Fig. 5C). By contrast, EMSA tests revealed clearly that AgaR2 protein bound the other three AgaR2‐binding palindromes in a dose‐dependent manner (Fig. 5D–F). Additionally, we also used *S. suis* AgaR2 protein to evaluate the function of the two AgaR‐binding sites in front of the *EF1809* locus of *E. faecalis* V583, a close relative of *S. suis*. As a result, AgaR2 physically interacted with the *EF1809* site 2, TTGTGGTTATAACCAGTT (Fig. 5H), but not the *EF1809* site 1, TTTATTGACAAAATAAAAAA (Fig. 5G) further validated the specificity of AgaR2 binding.

Different from the scenario with AgaR2, *S. suis* AgaR1 was only found to have the ability to interact with the predicted *SSU05_0448/9* site 1, TCTATTAATATACTAACACT (Fig. S3A). As anticipated from the comparative genomic analysis of the regulons, no interplay was observed between the AgaR2‐specific *SSU05_0448/9* site 2, TTGTGGTTATGTCCAGTA and *S. suis* AgaR1 protein (Fig. S3B), ruling out the possibility of cross‐regulation by AgaR2 and AgaR1. More importantly, the two putative AgaR1‐recognizable sites from *E. faecalis* V583 (referred to *EF814* site 1, AAATTCAATATATTAAGATA and *EF1809* site 1) were demonstrated to be functional, using gel shift assays with the *S. suis* AgaR1 protein (Fig. S3C and D).

### Regulatory roles of AgaR2 (AgaR1) in *Streptococcus* GalNAc utilization

**Figure 6:**
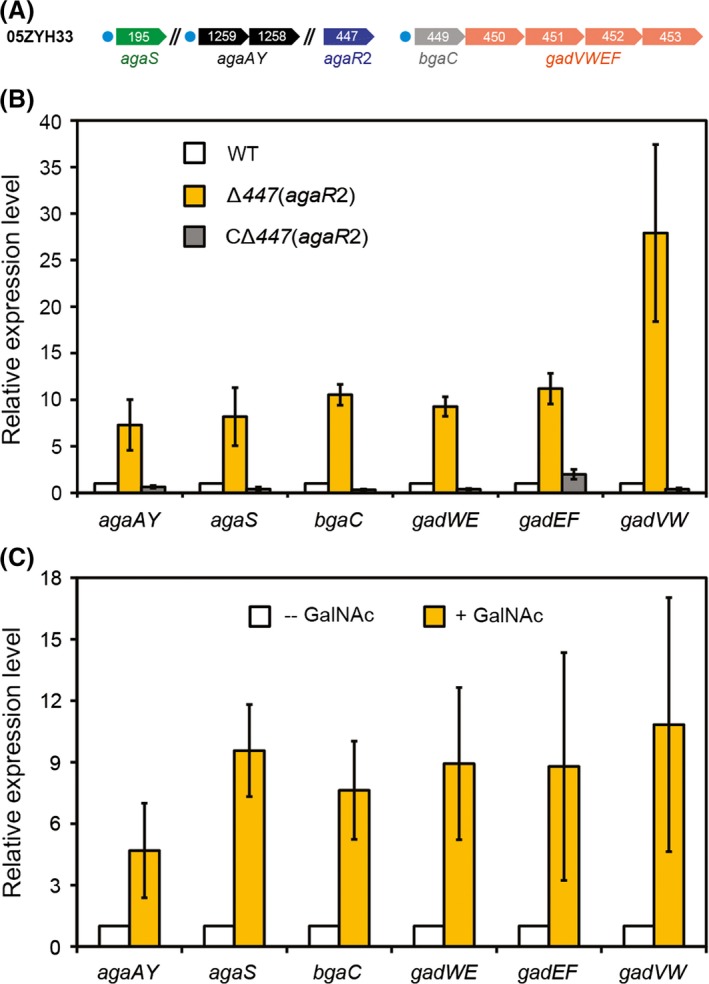
GalNAc/GalN utilization pathway is repressed by AgaR2 and induced by addition of GalNAc. (A) Schematic diagram for *agaR*2 and its cognate target genes.Circles denote the AgaR2‐recognizable sites, and the arrows represent the genes of the GalNAc utilization pathway. Note: SSU05_1259 and SSU05_1258 constitutes an operon *agaAY*, whereas the four ordered genes (SSU05_0449, SSU05_0450, SSU05_0451, and SSU05_0452) might form an operon of *bgaC*‐*gadVWEF*. (B) Real‐time qPCR assays for effects of *agaR*2 on expression profile of genes of GalNAc/GalN utilization pathway in *Streptococcus suis*. (C) Addition of GalNAc increases expression level of GalNAc/GalN utilization pathway‐encoding genes in *S. suis*.The data are expressed as averages ± SD, and error bars mean SD. No less than five independent experiments were carried out here. The minus sign denotes no addition of GalNAc into growth media, whereas the plus sign denotes addition of GalNAc in vitro. Note: *P* < 0.005. GalNAc, *N*‐acetyl‐d‐galactosamine; GalN, galactosamine; qPCR, quantitative PCR; SD, standard deviations.

Given the observation that AgaR2 efficiently binds to the promoter regions of the three genes/operons (*agaS* (*SSU05_0195*), *agaY* (*SSU05_1259*), and *bgaC* (*SSU05_0448*)) encoding GalNAc utilization pathway in *S. suis* (Figs. 5, 6A), we therefore attempted to elucidate its possible function in modulating *Streptococcus* GalNAc utilization machinery. The Δ*agaR*2 (Δ*447*) isogenic mutant was constructed, using an approach of homologous recombination, and its complementary strain CΔ*agaR*2 (CΔ*447*) was generated through the low‐copy plasmid pVA838‐borne expression of *agaR*2 gene (Table S1). We noted that the deletion of *agaR*2 does not affect its growth (Fig. S1A). Additionally, no obvious alteration of capsule was observed in the Δ *agaR*2 mutant in comparison with the WT strain 05ZYH33 (Fig. S1B and C). The qPCR‐based transcriptional analyses showed that removal of *agaR*2 gene gave at least five‐fold increment of *agaS* (*agaAY* and *bgaC‐gadVWEF* operon) expression (Fig. 6B). Whereas the transcription of the above target genes was restored to the level seen in its parental strain *S. suis* 05ZYH33 (Fig. 6B). Similar results were also observed in altered expression profile of the Δ*agaR*2 mutant revealed by the RNA‐Seq (e.g., expression of the three genes *SSU05_0195* (*agaS*), *SSU05_1259* (*agaA*), and *SSU05_1258* (*agaY*) are elevated upon removal of *agaR*2, Table 1). Therefore, it seemed true that AgaR2 is a functional repressor for the *Streptococcus* GalNAc utilization pathway. In contrast, we failed to visualize any significant alteration of the *bgaC* (*SSU05_0448*) expression level in the Δ*agaR*1 mutant in comparison with the WT strain and its complementary strain C*agaR*1 (not shown). The suggested possibility that *agaR*1 might play an uncovered role or be an evolutional relic with loss of physiological demand and/or advantage, in that (1) there is no in vivo regulatory role attributed to its protein product AgaR1 although it retains DNA‐binding activity to its own promoter, (2) the fact that all the other GalNAc metabolism‐related genes are without control by AgaR1 is due to the lack of the predicted AgaR1‐recognizable palindromes, (3) no physiological function can be present in GalNAc metabolism, even AgaR1 can autoregulate itself. Thus, our subsequent interests focused on AgaR2 regulator.

**Table 1:** RNA‐Seq assays for expression profile of genes encoding *N*‐acetyl‐d‐galactosamine utilization pathway

Altered expression profile				
Gene ID	Function	Ratio (Δ*agaR*2/WT)	Up/down regulation	*P*‐value
05SSU0195 (*agaS*)	Phosphosugar isomerase	6.20	Up	3.12836E‐261
05SSU1259 (*agaA*)	*N*‐acetylglucosamine‐6‐phosphate deacetylase	3.12	Up	6.71E‐98
05SSU1258 (*agaY*)	Tagatose 1,6‐diphosphate aldolase	2.98	Up	1.27E‐151

Considering the direct relevance of AgaR2 to GalNAc utilization in *S. suis*, it is reasonable to probe the possibility whether this regulator acts to be responsive to the presence of GalNAc. We thereby grew WT strain *S. suis* 05ZYH33 in the medium with/without the supplementation of GalNAc, and compared the expression profile of relevant target genes like *agaS*. As anticipated, the expression level of all the tested GalNAc catabolism pathway‐encoding genes (including *agaS* and *bgaC*) was given a 5‐ to 10‐fold increment upon the addition of 10 mmol/L GalNAc into growth conditions (Fig. 6C). Together, we concluded that the GalNAc/GalN utilization pathway is repressed mainly by the AgaR2 regulator, whereas induced by an addition of GalNAc.

### Contribution of AgaR2 (AgaR1) to bacterial pathogenesis

As we knew that amino sugars (GalN and/or GalNAc) constitute common residues for surface structure of various bacterial cell walls (Bernatchez et al. 2005; Freymond et al. 2006), we thus ambitiously reasoned that (1) such a bacterial surface structure might be involved in mutual communication/crosstalk between bacterial pathogens and the inhabited/infected hosts, (2) the maintenance and regulation of GalN/GalNAc catabolism is probably implicated into successful infections of some bacterial pathogens. To probe possible interference between GalN/GalNAc catabolism and the formation of virulence‐associated surface structures, we systemically employed two lines of approaches including cell lines‐based tests and infections of experimental animals.

**Figure 7:**
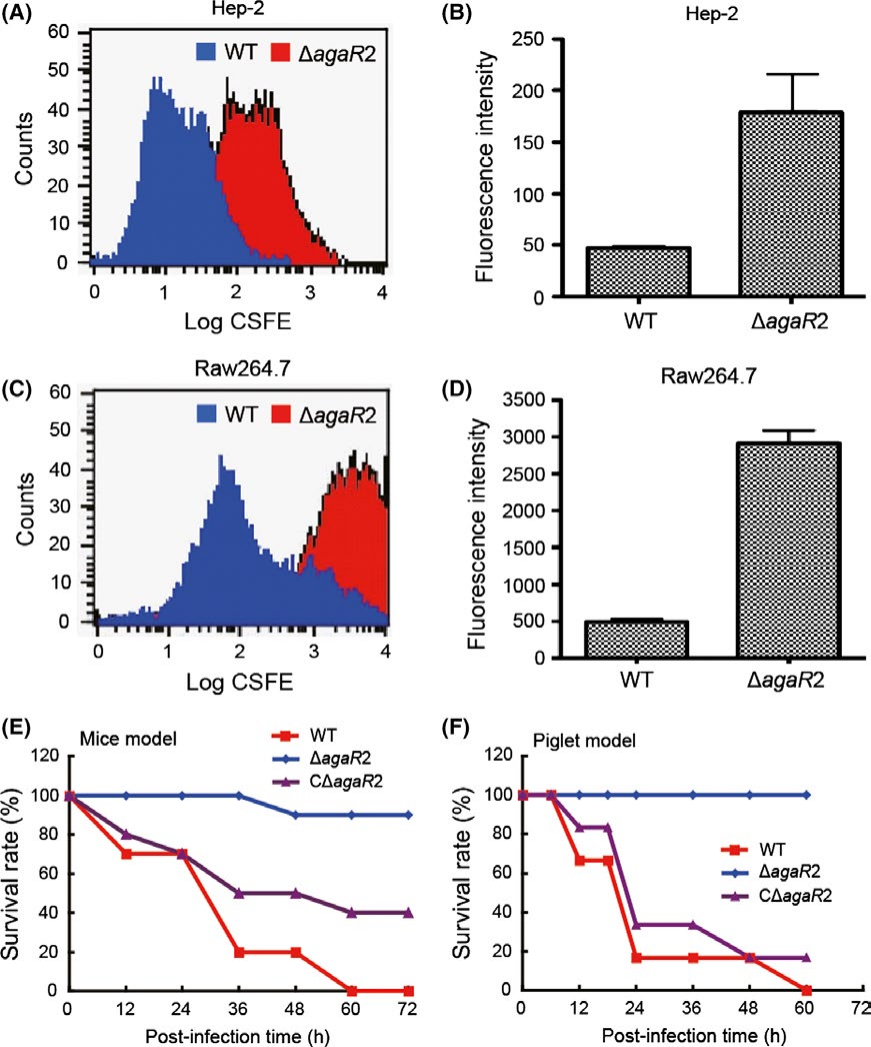
Role of AgaR2‐mediated regulation of GalNAc/GalN catabolism in bacterial virulence. (A) FACS‐based visualization for a role of *agaR*2 (ZYH05_0447) gene in adherence of *Streptococcus suis* 2 to Hep‐2 cells. (B) Quantitative analyses for effects of *agaR*2 deletion on adherence of *S. suis* 2 to Hep‐2 cells. (C) FACS‐based visualization for the relevance of *agaR*2 gene to the ability of bacterial anti‐phagocytosis against macrophage Raw264.7 cells. (D) Quantitative analyses for effects of *agaR*2 gene exerted on the ability of bacterial anti‐phagocytosis against macrophage Raw264.7 cells. (E) Evaluation for a role of *agaR*2 gene in bacterial virulence using infection model of mice. (F) Use of experimental infection of piglets to assay the relevance of *agaR*2 gene to bacterial pathogenesis. Note: *P* < 0.01. GalNAc, *N*‐acetyl‐d‐galactosamine; GalN, galactosamine; FACS, fluorescence‐activated cell sorting.

First, Hep‐2 cell line was used to evaluate the ability of bacterial adherence (Fig. 7A and B), and RAW264.7 macrophage was subjected to dissecting its capability of anti‐phagocytosis (Fig. 7C and D). Fluorescence‐activated cell sorting (FACS)‐based experiments elucidated that around threefold increment of bacterial adherence potential to Hep‐2 cells was given in the Δ*agaR*2 mutant relative to the WT 05ZYH33 strain (Fig. 7A and B). Furthermore, the deletion of *agaR*2 gene was found to give nearly fivefold increment of anti‐phagocytosis ability against RAW264.7 macrophage (Fig. 7C and D). This finding was in much similarity to scenarios seen with both Δ*neuB* and Δ*cps2B* mutants of *S. suis* 2 (note: the two genes are involved in bacterial surface architecture) (Feng et al. 2012). Thereby, we anticipated that the enhancement in abilities of bacterial attachment and anti‐phagocytosis might be partially due to the altered carbohydrates with GalN/GalNAc residues on bacterial cell wall surface and this kind of alteration could be attributed to the dysfunction in AgaR2‐mediated regulation of GalN/GalNAc utilization pathway. In contrast, no obvious difference between the Δ*agaR*1 mutant and its parental strain 05ZYH33 was observed on either bacterial adherence to Hep‐2 cell line or bacterial anti‐phagocytosis against RAW264.7 macrophage (not shown). In fact, such a different effect exerted by AgaR2 (and AgaR1) is not very surprising, in that only Aga2 (not AgaR1) evolved to possess a regulatory role in controlling GalNAc catabolism in *S. suis*.

Given the fact that not only the two mutants (Δ*neuB* and Δ*cps2B*) of *S. suis* 2 feature the potential of both increased adherence/anti‐phagocytosis, but also exhibit dramatically reduced virulence in infection models of both mice and piglets (Feng et al. 2012), it is of much interest to further probe a possible role of *agaR*2 in bacterial virulence. In the mice infection assays, all the 10 BALB/c (4‐week old, female) mice infected with the WT strain 05ZYH33 were sick shortly, and most of the rats died within 60 h (Fig. 7E). By contrast, mice of the negative control group inoculated with THB survived (not shown). In particular note, nearly all the mice infected with the Δ*agaR*2 mutant survived, while most of animals injected with the complementary strain CΔ*agaR*2 died within 3 days. To verify the results obtained from the above model of mice, we repeated the infection tests using the model of SPF‐piglets, natural hosts for *S. suis*. As a result, we observed that the six SPF‐piglets inoculated with WT virulent strain developed most of the typical disease symptoms (high fever, limping, swollen joints, etc.), and most of them died on day 1 (Fig. 7F). As expected, all the piglets infected with the Δ*agaR*2 mutant survived during the period of entire experiment. However, the re‐introduction of *agaR*2 gene into the Δ*agaR*2 mutant restored fully its strong virulence (Fig. 7F). Collectively, we believed that the prevalent regulator AgaR2 for GalN/GalNAc utilization pathway contributes to bacterial infectivity of *S. suis*, although we failed to note an apparent role of the other secondary regulator AgaR1 in bacterial pathogenicity (not shown).

## Discussion

The data shown here represents a first report that illustrated the genomic reconstruction of GalN/GalNAc catabolism pathway in *Streptococci* and *Firmicutes*. Different from the scenarios described in *E. coli* (Reizer et al. 1996) and *Shewanella* (Leyn et al. 2012), a significant variation of this pathway was proposed for *Streptococci* (Fig. 1 and Table S4). As you can see from the working model, AgaZ is absent in *S. suis*. It seemed likely that our continued genomic analyses pointed out that *S. suis* actually lacks any ortholog of this gene. Thereby, we speculated that the loss of AgaZ function in *S. suis* might be compensated by two other genes that are not in *aga* regulon: (1) SSU05_1041 (*lacC*2; tagatose 6‐phosphate kinase, EC 2.7.1.144), (2) SSU05_0543 (*pfk*, 6‐phosphofructokinase, EC 2.7.1.11). This hypothesis required further experimental verification.

Through extensive analyses for genomic contexts from *Firmicutes*, we dissected two GalN/GalNAc pathway‐specific regulators namely AgaR2 and AgaR1. The *agaR*1 gene is divergently transcribed into genes forming an operon and encoding a *β*‐galactosidase (BgaC) (Hu et al. 2014) and a PTS sugar uptake system (named *agaBCDE* that could be a GalN uptake system). The *agaR*2 gene in many *Streptococcus* species is located in front of the genes encoding the predicted galactosamine‐6‐phosphate isomerase and Tagatose 1,6‐diphosphate aldolase, two key enzymes in the AGA catablic pathway. In *S. suis*, the locus *SSU05_0448* is annotated to be *agaR*1, whereas *SSU05_0447* is referred to *agaR*2. Somewhat evolutionally distinct from the paradigm regulator (the *E. coli agaR* protein product) that belongs to the DeoR family of transcription factor, the two newly identified regulators AgaR2 and AgaR1 are classified into the GntR family of transcription repressors (Figs. 3, S1). Additionally, it seemed likely that the two GntR‐like regulators (AgaR2 and AgaR1) themselves fit into two different subgroups and might possess the varied preference in their DNA‐binding sites (Figs. 3, S4). The fact that AgaR2‐recognizable palindrome is predicted to be present in front of multiple target genes whereas AgaR1 has only one site located in its own promoter region (Fig. 2) raised the possibility that AgaR2 could have been evolved into a prevalent/major regulator for GalN/GalNAc utilization pathway (AgaR1 might be a minor/and even a cryptic regulator). Our in vitro gel shift assays validated the direct interaction between the AgaR2 (and/or AgaR1) protein and its binding sites (Figs. 5, S3). Given the facts that a significant regulated expression of GalN/GalNAc utilization‐related genes (such as *agaS*,* agaAY* , and *bgaC)* by AgaR2 was observed, however, no in vivo role could be attributed to AgaR1, we came to be more confident that AgaR2 relative to AgaR1 acts as a prevalent/leading player in modulating expression of genes encoding bacterial GalN/GalNAc catabolism in most *Firmicutes*. However, we did not obtain evidence for physiological ligand/molecular effector of AgaR2 repressor, although we observed that GalNAc does induce the expression of GalN/GalNAc catabolism‐related genes such as *agaS* (Fig. 6C).

The essence that amino sugars (GalN and/or GalNAc) are common components on the surface of bacterial cell walls (Bernatchez et al. 2005; Freymond et al. 2006) has driven us to test possible roles for the regulation of GalN/GalNAc catabolism in the infectivity of bacterial pathogens. As expected, an interference of GalN/GalNAc catabolism/utilization by disrupting *agaR*2 encoding a prevalent regulator, significantly altered abilities of both bacterial adherence to Hep‐2 cells and anti‐phagocytosis against RAW264.7 macrophage (Fig. 7). Given the fact that in this alteration we observed a similar scenario seen with the two virulence‐associated determinants (*neuB* and *cps2B*) with biological roles in constitution/development of bacterial surface architecture (Feng et al. 2012), we reasoned that regulation/maintenance of GalN/GalNAc catabolism pathway is essential for full virulence of *S. suis*. In fact, this proposal was subsequently proved in that the dysfunction in AgaR2‐mediated regulation impairs bacterial infectivity of *S. suis* in both mice and piglets (Fig. 7). Also, we are not surprised to establish the relevance of GalN/GalNAc catabolism to bacterial pathogenicity, in that the alteration of carbohydrates with GalN/GalNAc residues on bacterial cell surface interferes with the bacterial pathogen‐binding host cells.

Taken together, we are first to report the genomic reconstruction of the GalN/GalNAc utilization pathway in *S. suis* and its novel regulatory network with variations. More intriguingly, we revealed that the interference of GalN/GalNAc utilization pathway by the inactivation of the AgaR2 regulator attenuates greatly the bacterial infectivity of the zoonotic pathogen *S. suis*. Our finding might provide a metabolic basis for design of small molecule drugs (inhibitors)‐based therapeutics against *S. suis* infection through targeting the regulatory network of GalN/GalNAc utilization pathway.

## Conflict of Interest

None declared.

## Supporting information


**Table S1.** Strains and plasmids used in this study.
**Table S2.** DNA primers used in this study.
**Table S3.** AgaR regulons in Firmicutes.
**Table S4.** AgaR1 (AgaR2) binding sites.
**Figure S1.** Multiple sequence alignments of SSU05_0447 (AgaR2) with two other bacterial homologs. The three homologous proteins used here included *Bacillus subtilus* NagR (NC_018520.1), SSU05_0447 (AgaR2) of *Streptococcus suis* 05ZYH33 (NC_009442.1), and *Escherichia coli* AgaR (NC_007779.1). The program of ClustalW2 (http://www.ebi.ac.uk/Tools/clustalw2/index.html) was applied to conduct the multiple alignment of protein sequences, and the final output is generated by the ESPript 2.2 program (http://espript.ibcp.fr/ESPript/cgi-bin/ESPript.cgi). Identical residues are in white letters with a red background, similar residues are in red letters with a white background, varied residues are in black letters, and dots represent gaps. The predicted protein secondary structure is given on the top. Designations: NagR, *N*‐acetylglucosamine repressor; AgaR, acetyl‐galactosamine repressor; bs, *Bacillus subtilus*; ec, *E. coli*,* α*,* α*‐helix; *β*,* β*‐sheet; T, *β*‐turns/coils.
**Figure S2.** Purification, verification, and characterization of the AgaR1 (SSU05_0448) protein. (A) 12% SDS‐PAGE profile of the purified AgaR1 (SSU05_0448) protein from *Streptococcus suis*. (B) Western blot analyses for the N‐terminal 6x his tagged AgaR1 protein, using the anti‐6xHis tag primary antibody. The monomeric protein with expected size of ~30 kDa is indicated with an arrow, whereas the dimer form (~60 kDa) is highlighted with an asterisk. Designations: M, protein standard marker; WB, western blot. (C) Determination for the solution structure of the AgaR1 protein, using chemical cross‐linking assays. The chemical cross‐linker used here is ethylene glycol bis‐succinimidylsuccinate (EGS). The triangle on the top represents the addition of the EGS cross‐linker in varied concentrations (0.1, 0.2, 0.5, 1.0, 2.5, 5, 10, 20 *µ*mol/L in the right‐hand eight lanes [left to right]). The minus sign denotes no addition of EGS. The protein sample was separated with 12% SDS‐PAGE. The MS‐based identification of the purified AgaR1 protein with the solution structure of both monomer (in D) and dimer (in E). The tryptic peptides that match the AgaR1 protein are given in bold and under‐lined type.
**Figure S3.** Binding of AgaR1 to the predicted palindromes. The predicted AgaR1‐binding site (in A) of *SSU05_0448/9* gene bound AgaR1 (*SSU05_0448*) protein, whereas the AgaR2‐binding site (in B) of this locus does not interact with AgaR1 protein. (C) *Streptococcus suis* AgaR1 protein bound to the predicted AgaR1 site of *EF814* gene from *Enterococcus faecalis* V583. (D) *S. suis* AgaR1 protein bound to the putative AgaR1 site of *EF1809* gene from *E. faecalis* V583. The minus sign denotes no addition of AgaA1 protein. The protein levels of AgaR2 (on the right‐hand four lanes of each panel [left to right]) were 0.5, 1, 2, and 5 pmol. The protein samples were incubated with 0.2 pmol of the DIG‐labeled probe in a total volume of 15 *µ*L. A representative result from three independent gel shift assays (7% native PAGE) is given.
**Figure S4.** Phylogenetic analyses of phosphotransferase *system* (PTS). In total, the PTS system is classified into five sub‐groups, one of which is PTS‐V (highlighted in blue). Locus tag of PTS system is showed here for AgaC, SSU05_0451 is indicated in red.
