# Supplementary material for: Two novel regulators of N‐acetyl‐galactosamine utilization pathway and distinct roles in bacterial infections
Source: Microbiologyopen. 2015 Nov 5;4(6):983–1000. doi: 10.1002/mbo3.307 (PMC4694137; doi:10.1002/mbo3.307)
Supplement: Supplementary file 1 — Table S1. Strains and plasmids used in this study. Table S2. DNA primers used in this study. Table S3. AgaR regulons in Firmicutes. Table S4. AgaR1 (AgaR2) binding sites. Figure S1. Multiple sequence alignments of SSU05_0447 (AgaR2) with two other bacterial homologs. The three homologous proteins used here included Bacillus subtilus NagR (NC_018520.1), SSU05_0447 (AgaR2) of Streptococcus suis 05ZYH33 (NC_009442.1), and Escherichia coli AgaR (NC_007779.1). The program of ClustalW2 (http://www.ebi.ac.uk/Tools/clustalw2/index.html) was applied to conduct the multiple alignment of protein sequences, and the final output is generated by the ESPript 2.2 program (http://espript.ibcp.fr/ESPript/cgi-bin/ESPript.cgi). Identical residues are in white letters with a red background, similar residues are in red letters with a white background, varied residues are in black letters, and dots represent gaps. The predicted protein secondary structure is given on the top. Designations: NagR, N‐acetylglucosamine repressor; AgaR, acetyl‐galactosamine repressor; bs, Bacillus subtilus; ec, E. coli, α, α‐helix; β, β‐sheet; T, β‐turns/coils. Figure S2. Purification, verification, and characterization of the AgaR1 (SSU05_0448) protein. (A) 12% SDS‐PAGE profile of the purified AgaR1 (SSU05_0448) protein from Streptococcus suis. (B) Western blot analyses for the N‐terminal 6x his tagged AgaR1 protein, using the anti‐6xHis tag primary antibody. The monomeric protein with expected size of ~30 kDa is indicated with an arrow, whereas the dimer form (~60 kDa) is highlighted with an asterisk. Designations: M, protein standard marker; WB, western blot. (C) Determination for the solution structure of the AgaR1 protein, using chemical cross‐linking assays. The chemical cross‐linker used here is ethylene glycol bis‐succinimidylsuccinate (EGS). The triangle on the top represents the addition of the EGS cross‐linker in varied concentrations (0.1, 0.2, 0.5, 1.0, 2.5, 5, 10, 20 µmol/L in the right‐hand eigh [file MBO3-4-0983-s001.docx]

**Supplemental Information**

**Supplemental tables**

**Table S1** Strains and plasmids used in this study

| **Strains or plasmids** | | **Relevant characteristics** | **Refs or origins** |
| --- | --- | --- | --- |
| *E. coli* strains | | | |
| Topo10 | | A cloning host of *E. coli* (F^-^, Δ*lac*X74) | Invitrogen |
| BL21(DE3) | | An expression host of *E. coli* | Lab stock |
| FYJ356 | | BL21(DE3) carrying pET28-*448* | This study |
| FYJ536 | | BL21(DE3) carrying pET28-*447* | This study |
| *S. suis* strains | |  |  |
| 05ZYH33 | | The wild type of Chinese virulent *S. suis* 2 | ([1-3](#_ENREF_1)) |
| Δ*SSU05_0447* | | The mutant strain of 05ZYH33 in which the *SSU05_0447* gene is inactivated | This study |
| CΔ*SSU05_0447* | | The complemented strain of the mutant Δ*SSU05_0447* carrying a plasmid-borne *SSU05_0447* gene | This study |
| Δ*SSU05_0448* | | The mutant strain of 05ZYH33 in which the gene *SSU05_0448* is inactivated | This study |
| CΔ*SSU05_0448* | | The complementary strain of the mutant Δ*SSU05_0448* carrying a plasmid-borne *SSU05_0448* gene | This study |
| Plasmids |  | |  |
| pCR2.1 | High copy Topo-cloning vector, Amp^R^Km^R^ | | Invitrogen |
| pET28(a) | Commercial T7-driven expression vector, Km^R^ | | Novagen |
| pET28*-447* | pET28 carrying *SSU05_0447*, Km^R^ | | This study |
| pET28*-448* | pET28 carrying *SSU05_0448*, Km^R^ | | This study |
| pUC19 | A cloning vector with high copy, Amp^R^ | | Invitrogen |
| pUC19-Spc | pUC19 carrying the Spc^R^ cassette | | This study |
| pUC*::447* | A knockout plasmid of the gene *SSU05_0447* | | This study |
| pUC*::448* | A recombinant plasmid to knockout the gene *SSU05_0448* | | This study |
| pVA838 | A low copy shuttle plasmid that replicates in *E. coli* and *Streptococcus suis* | | ([4](#_ENREF_4)) |
| pVA838*-447* | pVA838 encoding the gene *SSU05_0447* | | This study |
| pVA838*-448* | pVA838 encoding the gene *SSU05_0448* | | This study |
| Cell lines |  | |  |
| Raw 264.7 | Mouse macrophage cell | | ATCC TIB-71, ([5](#_ENREF_5), [6](#_ENREF_6)) |
| Hep-2 | Larynx carcinoma epithelial cell | | CCTCC GDC004, ([5](#_ENREF_5), [6](#_ENREF_6)) |

^a^Spc^R^, spectinomycin resistance; Amp^R^, ampicillin resistance ; Kan^R^, kanamycin resistance.

**Table S2** DNA primers used in this study

| **Primers** | **Primer sequences** |
| --- | --- |
| *SSU05_0447*-F (BamHI) | 5'-CG *GGATCC* ATG TGG GTT AGA AAG GAG G-3’ |
| *SSU05_0447*-R (XhoI) | 5'-CCG *CTCGAG* CTA ATA TCC CCG TTG ATG GGT A-3' |
| *SSU05_0448*-F (BamHI) | 5'-CG *GGATCC* ATGAAAGTACCGAAGTACCAA-3’ |
| *SSU05_0448*-R (SalI) | 5'-CCG *GTCGAC* TTA GCG ATG GTT GGC TGT AA-3' |
| *SSU05_0447*-P-F | 5'-CTG GCT GTA GAT CTA AGT AG-3' |
| *SSU05_0447*-P-R | 5'-CTC CTC CTT TCT AAC CCA CA-3' |
| 0447L-1(EcoRI) | 5’-GAATTCGATCGGATTCAGGCTGTCC-3' |
| 0447L-2 (BamHI) | 5’-GGATCCAAGCTTACAAATCTATTTTAC-3’ |
| 0447R-1 (SalI) | 5’-GTCGAC TAGAATCAATACTCTTCG-3’ |
| 0447R-2 (HindIII) | 5’-AAGCTT CTTCTTTGTGGTTATGTCCAGTAC-3’ |
| 0447In1 | 5’-GCGTGAGTTGACACAGGTTTATGG-3’ |
| 0447In2 | 5’-CACTGGACTATTGCTTGGAATTCC-3’ |
| 0447Out1 | 5’-GAAGATGATTTAATCGCCAAGGAA-3’ |
| 0447Out2 | 5’-TGAATCGCGCCTGATAAAATCTTA-3’ |
| C0447-F (BamHI) | 5’-GGATCCCCTTGCTTTAGCTTGAAGAGC-3’ |
| C0447-R (SalI) | 5’-GTCGACCAAGGCTCTTGACAACGAATA-3’ |
| 0448L-1(SacI) | 5’-GAGCTCATCCCTCAACAATCTTGGTAAT -3’ |
| 0448L-2(BamHI) | 5’-GGATCCACCCAATAACAATCAAAAGTAG -3’ |
| 0448R-1(SalI) | 5’-GTCGACAACTTATTATACTAAATTAGTT-3’ |
| 0448R-2(SphI) | 5’-GCATGCCATGTACAGATTCAAGGAGCC-3’ |
| 0448In1 | 5’-CCATCACTTCACCGCTAGTACT-3’ |
| 0448In2 | 5’-TTACGTGAACGAGATACAAAGG-3’ |
| 0448Out1 | 5’-CGTCGTGGTAAGATTACGCTGG-3’ |
| 0448Out2 | 5’-TACAAGCGGTTCTGCATACTCT-3’ |
| C0448-F(BamHI) | 5’-GGATCCCCAGTTCTATCTTCGGCT-3’ |
| C0448-R(SalI) | 5’-CGTCGACCTTTGTGGTTATGTCCAGT-3’ |
| *SSU05_0195*-site-F | 5’-TATAAGAAA**AACTGGTTATAACCAGAA**ATCCCGGGG-3’ |
| *SSU05_0195*-site-R | 5’-CCCCGGGAT**TTCTGGTTATAACCAGTT**TTTCTTATA-3’ |
| *SSU05_0447*-site-F | 5’-GATGAGGTA**ACCTTGCTTTAGCTTGAA**GAGCTGGTA-3’ |
| *SSU05_0447*-site-R | 5’-TACCAGCTC**TTCAAGCTAAAGCAAGGT**TACCTCATC-3’ |
| *SSU05_0448/9*-site2-F | 5’-AACACTTCT**TTGTGGTTATGTCCAGTA**CTTTTTAAA-3’ |
| *SSU05_0448/9*-site2-R | 5’-TTTAAAAAG**TACTGGACATAACCACAA**AGAAGTGTT-3’ |
| *SSU05_1259*-site-F | 5’-ATATTTGGT**ATATGGTTATAACCAGTT**TGATAAGTA-3’ |
| *SSU05_1259*-site-R | 5’-TACTTATCA**AACTGGTTATAACCATAT**ACCAAATAT-3’ |
| *SSU05_0448*-site1-F | 5’-TTCATCAAC**TAATTTAGTATAATAAGTTG**TGGAAAGGA-3’ |
| *SSU05_0448*-site1-R | 5’-TCCTTTCCA**CAACTTATTATACTAAATTA**GTTGATGAA-3’ |
| *agaAY*-F (321 bp) | 5’-GTGGTAGTTTGGCAGGTTCTATC-3’ |
| *agaAY*-R | 5’-GCTGTTTGACAAGCGAGTCATG-3’ |
| *agaS*-F (324 bp) | 5’-GCCAACTACCTCAACCAAGTC-3’ |
| *agaS*-R | 5’-GTAAGAACCTGTCATGGCAAAG-3’ |
| *agaR*1*-F* (327 bp) | 5’-GCGATGGTTGGCTGTAATTTC-3’ |
| *agaR*1*-R* | 5’-GACACTCCATACATCTTCCAC-3’ |
| *bgaC*-F (298 bp) | 5’-GTCCATCCTGATGATTGGTAC-3’ |
| *bgaC*-R | 5’-CATCTAAGTGCTGCAAGTACAC-3’ |
| *gadVW*-F (295 bp) | 5’-GGGAATATGTCTCAAACTCCG-3’ |
| *gadVW*-R | 5’-GCTGACGATACAATTGTCAGC-3’ |
| *gadWE*-F (320 bp) | 5’-GGTTTCGCTCTTACAGCTATG-3’ |
| *gadWE*-R | 5’-GTTGGAAAGTGAACAAGCTACG-3’ |
| *gadEF*-F (319 bp) | 5’-CAG GAT TTG TCT TCT GGC TTC-3’ |
| *gadEF*-R | 5’-CAACAAGTCTGCGAAGACAAC-3’ |
| *EF1809*-site1-F | 5’-TTAATACTT**TTTATTGACAAAATAAAAAA**AACCACCGT-3’ |
| *EF1809*-site1-R | 5’-ACGGTGGTT**TTTTTTATTTTGTCAATAAA**AAGTATTAA-3’ |
| *EF1809*-site2-F | 5’-ACTATTGGT**TTGTGGTTATAACCAGTT**AGGGTAAAG-3’ |
| *EF1809*-site2-R | 5’-CTTTACCCT**AACTGGTTATAACCACAA**ACCAATAGT-3’ |
| *EF0814/5*-site1-F | 5’-CAAAAAAAT**AAATTCAATATATTAAGATA**TTAGGTTTAC-3’ |
| *EF0814/5*-site1-R | 5’-GTAAACCTAA**TATCTTAATATATTGAATTT**ATTTTTTTG-3’ |

The restriction sites are underlined and the putative AgaR (or AgaR2) binding sites are in bold.

**Supplemental Figures**


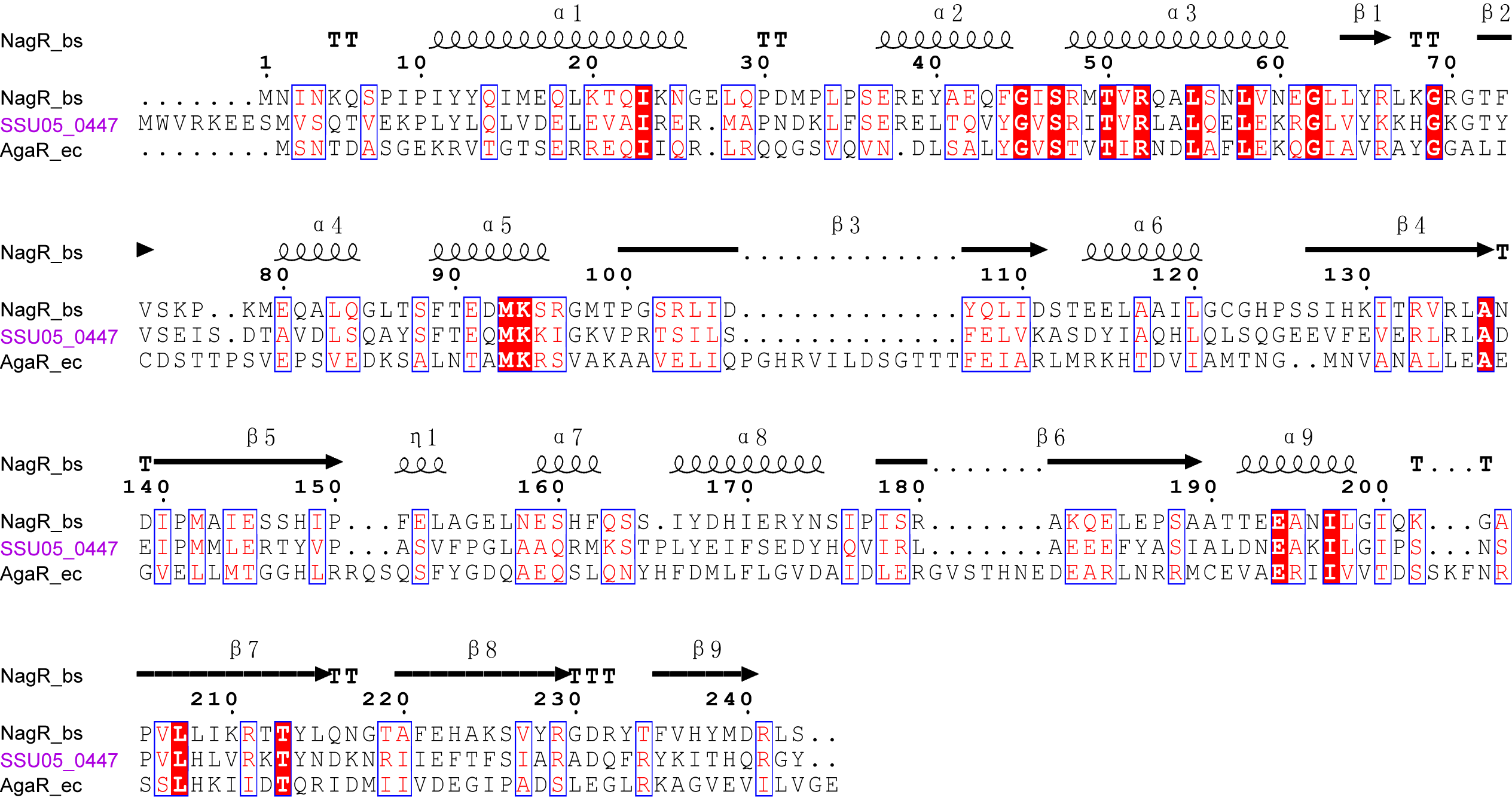


**Fig.S1** Multiple sequence alignments of SSU05_0447 (AgaR2) with two other bacterial homologues

The three homologous proteins used here included *Bacillus subtilus* NagR (NC_018520.1), SSU05_0447 (AgaR2) of *S. suis* 05ZYH33 (NC_009442.1), and *E. coli* AgaR (NC_007779.1).

The program of ClustalW2 (<http://www.ebi.ac.uk/Tools/clustalw2/index.html>) was applied to conduct the multiple alignment of protein sequences, and the final output is generated by the ESPript 2.2 program (<http://espript.ibcp.fr/ESPript/cgi-bin/ESPript.cgi>). Identical residues are in white letters with a red background, similar residues are in red letters with a white background, varied residues are in black letters, and dots represent gaps. The predicted protein secondary structure is given on the top.

Designations: NagR, N-acetylglucosamine repressor; AgaR, acetyl-galactosamine repressor; bs, *Bacillus subtilus*; ec, *E. coli*, α, α-helix; β, β-sheet; T, β-turns/coils.


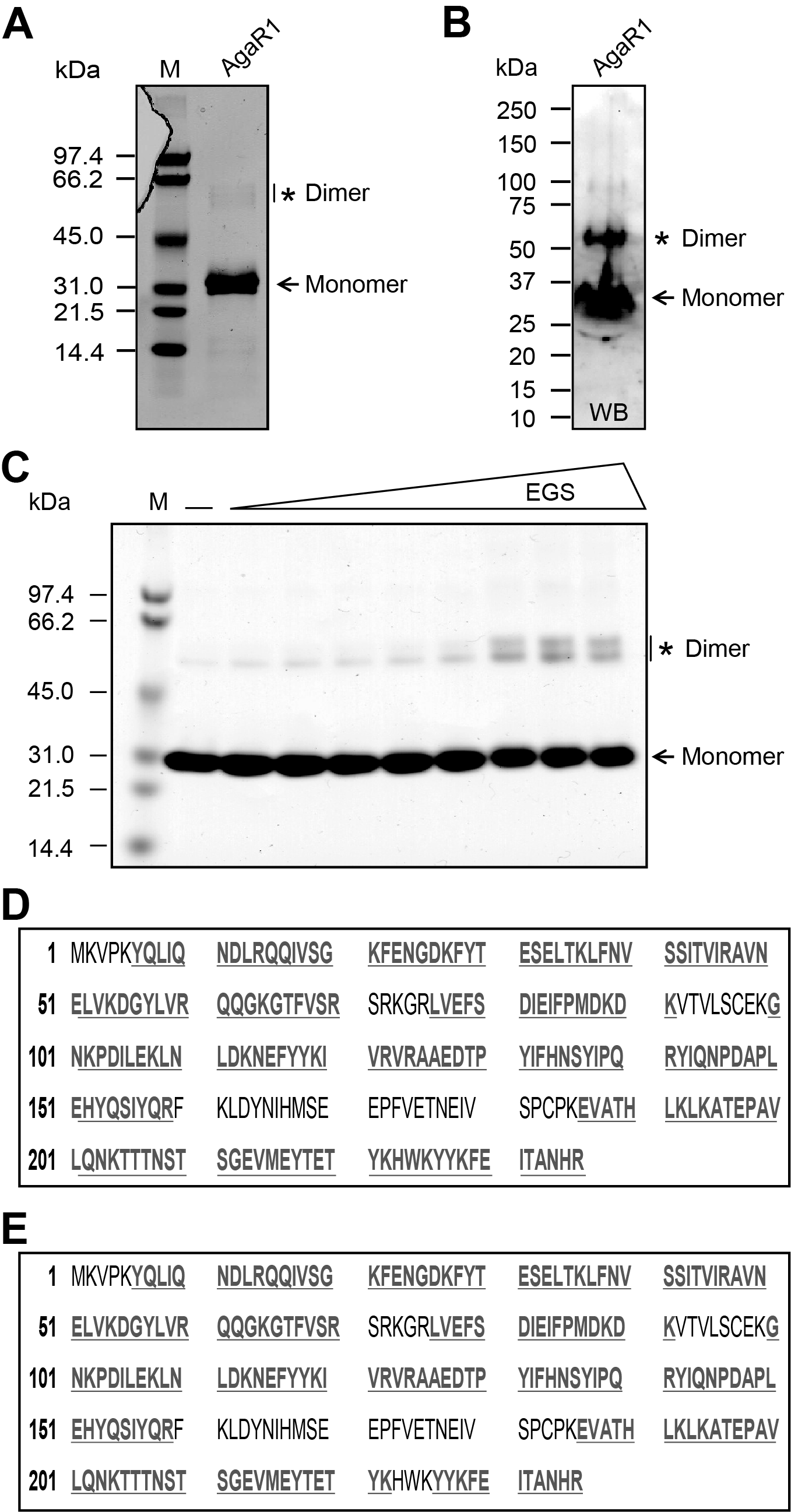


**Fig.S2** Purification, verification, and characterization of the AgaR1 (SSU05_0448) protein

**A.** 12% SDS-PAGE profile of the purified AgaR1 (SSU05_0448) protein from *S. suis*

**B.** Western blot analyses for the N-terminal 6x his tagged AgaR1 protein using the anti-6xHis tag primary antibody

The monomeric protein with expected size of ~30 kDa is indicated with an arrow, whereas the dimer form (~60 kDa) is highlighted with an asterisk.

Designations: M, protein standard marker; WB, western blot.

**C.** Determination for the solution structure of the AgaR1 protein using chemical cross-linking assays

The chemical cross-linker used here is ethylene glycol bis-succinimidylsuccinate (EGS). The triangle on the top represents the addition of EGS cross linker in varied concentrations (0.1, 0.2, 0.5, 1.0, 2.5, 5, 10, 20 µM in the right-hand eight lanes [left to right]). Minus sign denotes no addition of EGS. The protein sample was separated with 12 % SDS-PAGE.

MS-based identification of the purified AgaR1 protein with the solution structure of both monomer (in **Panel D**) and dimer (in **Panel E**)

The tryptic peptides that match the AgaR1 protein are given in bold and under-lined type.


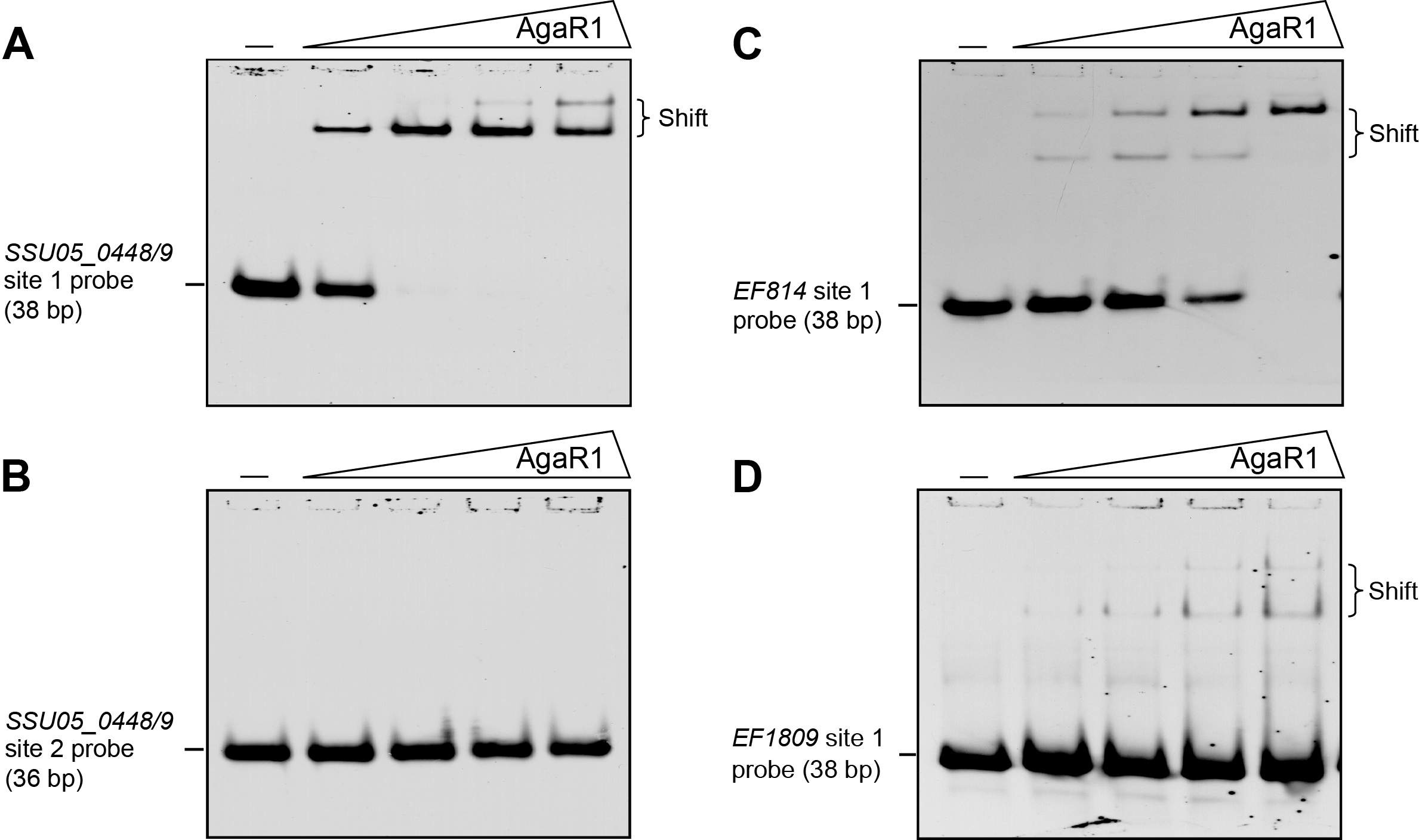


**Fig.S3** Binding of AgaR1 to the predicted palindromes

The predicted AgaR1-binding site (in **Panel A**) of *SSU05_0448/9* gene bound AgaR1 (*SSU05_0448*) protein, whereas the AgaR2-binding site (in **Panel B**) of this locus does not interact with AgaR1 protein.

**C.** *S. suis* AgaR1 protein bound the predicted AgaR1 site of *EF814* gene from *Enterococcus faecalis* V583

**D.** *S. suis* AgaR1 protein bound the putative AgaR1 site of *EF1809* gene from *Enterococcus faecalis* V583

The minus sign denotes no addition of AgaA1 protein. The protein levels of AgaR2 (in the right hand four lanes of each panel [left to right]) were 0.5, 1, 2 and 5 pmol. The protein samples were incubated with 0.2 pmol of DIG-labeled probe in a total volume of 15 µl. A representative result from three independent gel shift assays (7% native PAGE) is given.


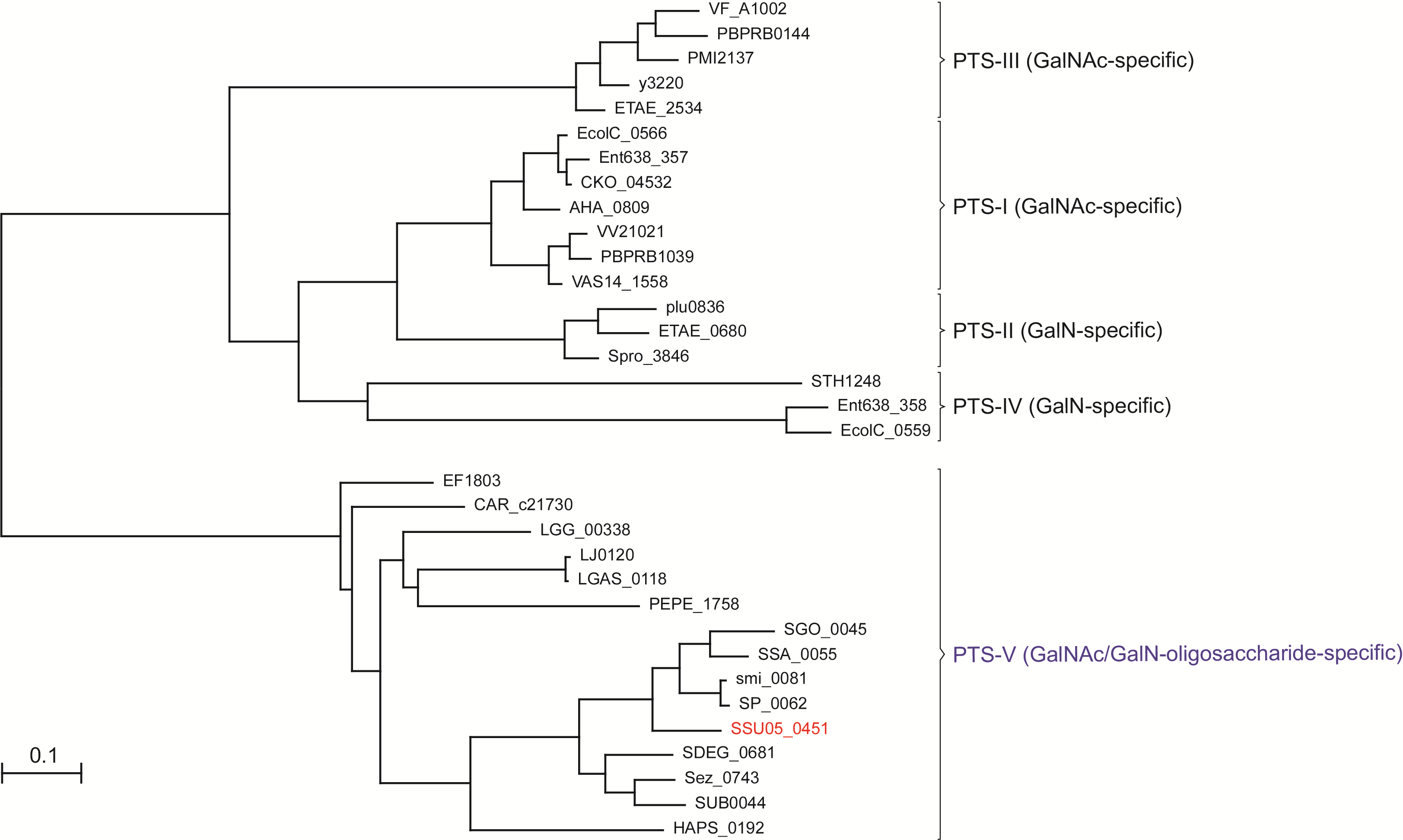


**Fig.S4** Phylogenetic analyses of phosphotransferase system (PTS)

In total, PTS system is classified into 5 sub-groups, one of which is PTS-V (highlighted in blue). Locus tag of PTS system is showed here for AgaC, SSU05_0451 is indicated in red.**Supplemental References**

1. Chen C*, et al.* (2007) A glimpse of streptococcal toxic shock syndrome from comparative genomics of *S. suis* 2 Chinese isolates. *PloS one* 2(3):e315.

2. Feng Y*, et al.* (2008) Functional definition and global regulation of Zur, a zinc uptake regulator in a *Streptococcus suis* serotype 2 strain causing streptococcal toxic shock syndrome. *Journal of bacteriology* 190(22):7567-7578.

3. Tang J*, et al.* (2006) Streptococcal toxic shock syndrome caused by *Streptococcus suis* serotype 2. *PLoS medicine* 3(5):e151.

4. Romero DA, Slos P, Robert C, Castellino I, & Mercenier A (1987) Conjugative mobilization as an alternative vector delivery system for lactic *streptococci*. *Applied and environmental microbiology* 53(10):2405-2413.

5. Feng Y*, et al.* (2012) Attenuation of *Streptococcus suis* virulence by the alteration of bacterial surface architecture. *Scientific reports* 2:710.

6. Hu D*, et al.* (2014) The β-galactosidase (BgaC) of the zoonotic pathogen *Streptococcus suis* is a surface protein without the involvement of bacterial virulence. *Scientific reports* 4:4140.
